# *Pfcyp51* exclusively determines reduced sensitivity to 14α-demethylase inhibitor fungicides in the banana black Sigatoka pathogen *Pseudocercospora fijiensis*

**DOI:** 10.1371/journal.pone.0223858

**Published:** 2019-10-17

**Authors:** Pablo Chong, Aikaterini-Eleni Vichou, Henk J. Schouten, Harold J. G. Meijer, Rafael E. Arango Isaza, Gert H. J. Kema

**Affiliations:** 1 ESPOL Polythecnic University, Escuela Superior Politécnica del Litoral, ESPOL, Centro de Investigaciones Biotecnológicas del Ecuador, Laboratorio de Fitopatología, Guayaquil, Ecuador; 2 Laboratory of Phytopathology, Wageningen University and Research, The Netherlands, Wageningen, the Netherlands; 3 Escuela de Biociencias, Faculta de Ciencias, Universidad Nacional de Colombia -Sede Medellín (UNALMED), Medellín, Colombia; 4 Unidad de biotecnología (UNALMED-CIB), Corporación para Investigaciones Biológicas, Medellín, Colombia; Korea University, REPUBLIC OF KOREA

## Abstract

The haploid fungus *Pseudocercospora fijiensis* causes black Sigatoka in banana and is chiefly controlled by extensive fungicide applications, threatening occupational health and the environment. The 14α-Demethylase Inhibitors (DMIs) are important disease control fungicides, but they lose sensitivity in a rather gradual fashion, suggesting an underlying polygenic genetic mechanism. In spite of this, evidence found thus far suggests that *P*. *fijiensis cyp51* gene mutations are the main responsible factor for sensitivity loss in the field. To better understand the mechanisms involved in DMI resistance, in this study we constructed a genetic map using DArTseq markers on two F1 populations generated by crossing two different DMI resistant strains with a sensitive strain. Analysis of the inheritance of DMI resistance in the F_1_ populations revealed two major and discrete DMI-sensitivity groups. This is an indicative of a single major responsible gene. Using the DMI-sensitivity scorings of both F1 populations and the generation of genetic linkage maps, the sensitivity causal factor was located in a single genetic region. Full agreement was found for genetic markers in either population, underlining the robustness of the approach. The two maps indicated a similar genetic region where the *Pfcyp51* gene is found. Sequence analyses of the *Pfcyp51* gene of the F_1_ populations also revealed a matching bimodal distribution with the DMI resistant. Amino acid substitutions in *P*. *fijiensis* CYP51 enzyme of the resistant progeny were previously correlated with the loss of DMI sensitivity. In addition, the resistant progeny inherited a *Pfcyp51* gene promoter insertion, composed of a repeat element with a palindromic core, also previously correlated with increased gene expression. This genetic approach confirms that *Pfcyp51* is the single explanatory gene for reduced sensitivity to DMI fungicides in the analysed *P*. *fijiensis* strains. Our study is the first genetic analysis to map the underlying genetic factors for reduced DMI efficacy.

## Introduction

The dothideomycete fungus *Pseudocercospora fijiensis* (previously *Mycosphaerella fijiensis*) is the causal agent of black Sigatoka, a major global threat to banana crops that is responsible for serious economic losses in banana production and provokes major negative environmental impacts due to current control strategies [1]. Contemporary disease control is mainly achieved by the application of systemic fungicides of which the most commonly used fungicides belong to the 14α-Demethylase Inhibitors (DMIs) group. DMI are single target fungicides, hence, sensitive to resistance development. Fungicide application frequencies for black Sigatoka management are extensive and DMIs are important constituents of the spray schedules, which have not only serious negative environmental and social impacts, but also contribute to the development of resistance in the pathogen populations [2–4]. In general, most microorganisms adapt to fungicides by the selection of individuals with modulated genetic information. Commonly observed genetic mechanisms resulting in reduced DMI sensitivity in *P*. *fijiensis* are point mutations in and overexpression of 14α-demethylase that is encoded by the *Pfcyp51* gene [5–10].

Abrupt loss of fungicide efficacy in the field is usually considered to be monogenic, resulting from mutations in a single major gene. As a result, the pathogen subpopulation carrying the mutation(s) becomes dominant and higher fungicide concentrations do not enable improved disease management, also indicated as qualitative resistance. The resistance to strobilurins in various plant pathogenic fungi, including *P*. *fijiensis*, illustrates this observation [11]. In contrast, quantitative and hence, gradually shifting reduced sensitivities are enabled by the interaction of a number of different genes [12]. DMI resistance mechanisms in fungi have a quantitative polygenic nature. In *Candida albicans*, *Aspergillus fumigatus* and *Zymoseptoria tritici* DMI resistance involves modification of sterol biosynthesis and increased expression of membrane transporters, e.g. ATP-binding cassette transporters and major facilitators, resulting in increased fungicide efflux that leads to reduced efficacy [13, 14]. All current evidence in *P*. *fijiensis* points to *Pfcyp51* as a major factor responsible for reduced sensitivity to DMIs. However, the loss of sensitivity to DMIs in the field has been gradual in nature [3, 5] and a recent study revealed extraordinary high EC_50_ values in some strains, questioning whether changes in the *Pfcyp51* gene are the only underlying genetic mechanism [15]. Hence, additional quantitative genetic components may exist that directly or indirectly modulate resistance.

*P*. *fijiensis* has a bipolar heterothallic mating system [16], which facilitates genetic studies by crossing strains with opposite mating types [11, 17]. Recently, a genetic linkage map for *P*. *fijiensis* was generated to support genome assembly, but specific mapping studies on fungal characteristics have not been accomplished [18]. The aim of the present study was to unravel the genetic basis for reduced sensitivity towards DMI’s fungicides in *P*. *fijiensis* by objective genetic mapping using Diversity Array Technology (DArTs) markers that also were used to generate a new genome assembly. Contrary to our expectations, progeny analyses provided strong evidence that DMI resistance in *P*. *fijiensis* is solely based on *Pfcyp51* modulation and not on other previously reported mechanisms such as increased efflux [14, 19, 20]. Despite increasing and accumulating data on fungicide resistance in fungal human and plant pathogens [4, 14, 21–27], our study is the first genetic analysis to map the underlying genetic factors for reduced DMI efficacy.

## Materials and methods

### Ethics statement

The strains CaM10_16, CaM10_6, CaM7_19, CaM7_10 and CaM10_21 were obtained after discharging ascospores form naturally infected leaves from the Cartagena plantation, Costa Rica, which were sent by the head of the phytopathology department Mr. Mauricio Guzmán MSc. He obtained permission to send these infected leaves to Wageningen University for scientific studies He is acknowledged for this support.

The strain Bo_1 was obtained after discharging ascospores form naturally infected leaves from banana plants at Bohol, Philippines, which were collected by commercial company Unifrutti through their employee Mrs. Lolit Bacus. She obtained permission to send this infected leaf to Wageningen University for scientific studies. She is acknowledged for this support.

The field collection of the leaves did not involve endangered species. *Pseudocercospora fijiensis* is a globally occurring pathogen of banana plants, both in plantation as well as in weedy banana plants occurring in the wild.

### Fungal isolation

Banana leaves with black Sigatoka symptoms were collected from untreated field plots on the island of Bohol, Philippines, and from the commercial Cartagena plantation in Costa Rica that is weekly sprayed with fungicides [15]. Infected leaf pieces (~2x3 cm) with mature necrotic lesions were retrieved and stapled to a circular 90 mm diameter filter paper (Whatman 113, Little Chalfont, UK). Filter papers containing four or five leaf pieces were incubated for 48 hours in humid chambers (sealed plastic container with humid cotton) and subsequently soaked in water for five minutes. The excess of water was blotted with paper towel and the filter papers were placed on the lid of inverted petri dishes filled with 1% water agar. The drop in relative humidity facilitates the discharge of ascospores and single spore strains were recovered with a needle after one-night incubation at 4°C in a refrigerator and transferred to Petri dishes with potato dextrose agar (PDA) medium that were incubated at 27°C in the dark for three weeks.

### Inoculum preparation

To prepare inoculum, a piece of mycelium (~0.5 cm^2^) from a mono-ascosporic *P*. *fijiensis* colony (three to four weeks old) grown on PDA was blended for 20 seconds at 6,000 rpm in an Ultra Turrax Tube Drive machine using a sterile DT-20 tube (Tube with rotor stator element, IKA, Staufen, Germany) in 15 ml of distilled water [28]. The mycelial fragments were filtered through a Steriflip Vacuum-driven Filtration System (100 μm, Millipore, Billerica, USA) and counted with a Kova glasstic slide 10 with a grids coverslip microscope slide (Kova, California, USA) and the suspensions were diluted to a final concentration of approximately 5x10^5^ mycelial fragments per ml.

### Microtiter experiments and analyses

From the abovementioned mycelium solution, a 50 μl aliquot was transferred to each well of a 96-well microtiter plate (Corning 96-well Flat Bottom Transparent Polystyrol uncoated, Corning, USA) that were filled with 200 μl potato dextrose broth (PDB) medium with antifungal compounds. Seven compound concentrations were tested with two technical repetitions per strain. All experiments were repeated three times. The samples were incubated in the dark at 27°C for 10 days to allow the mycelium to grow. Subsequently, the microtiter plates were analyzed in the Infinite® 200 PRO machine branch (TEKAN, Switzerland) at room temperature (~20°C), without cover, at a wavelength of 690 nm with multiple reads per well in a 5x5 circle-filled form to determine mycelium proliferation by optical density. The bandwidth was 9 μm with five flashes per read that started 1 mm from the well wall to prevent border effects.

### Fungicide compounds

The fungicides propiconazole, difenoconazole and epoxiconazole were provided by Syngenta Crop Protection AG (Basel, Switzerland), were of technical grade quality and maintained as stock solution in DMSO (propiconazole and difenoconazole at 50,000x and epoxiconazole at 20,000x). The final testing concentrations for all compounds were 0; 0.004; 0.016; 0.04; 0.16; 0.64; 2.56 and 10.24 mg.l^-1^ with 1% DMSO. The sensitivity ranges for the compounds were established by calculating the 50% effective concentration (EC_50_) by plotting the growth profiles based on OD readings. Monotone regression spline functions [29] were applied to fit the curve profiles using GenStat 18th Edition software (VSN International, Hemel Hempstead, UK). The EC_50_ thresholds for categorizing *P*. *fijiensis* strains as either DMI resistant or sensitive were arbitrary chosen based on the cluster analysis of the Least Standard error of the Differences (LSD) of the log_2_ EC_50_ individual means in each population. The selected EC_50_ thresholds were: resistant strains >1 mg.l^-1^ and sensitive strains ≤ 0.2 mg.l^-1^.

### Crosses between sensitive and resistant *P*. *fijiensis* strains

Five DMI resistant mono-ascosporic *P*. *fijiensis* field strains from Costa Rica (CaM10_16, CaM10_6, CaM7_19, CaM7_10 and CaM10_21), maintained in the Plant Research International collection were selected for crosses with the mono-ascosporic wild type strain Bo_1 (Bohol, Philippines, Table 1). The strains were crossed shortly after the first sensitivity assay to avoid loss of sexual fitness as experienced in numerous other tries for developing *P*. *fijiensis* mapping populations (no more than two sub-cultivation steps). The mating type locus (*mat)* configuration was determined using the *mat1-1* primers *Mat1F* (5’-CATGAGCACGCTGCAGCAAG-3’) and *Mat1R* (5’-GTAGCAGTGGTTGACCAGGTCAT-3’) and the *mat1-2* primers *Mat2F* (5’-GGCGCTCCGGCAAATCTTC-3’) and *Mat2R* (5’-CTTCTCGGATGGCTTGCGTG-3’) [30]. The PCR reaction was performed using Roche Taq DNA polymerase with a standard mix containing 10 ng of gDNA according to the following protocol: 94°C for 4 min., then 30 cycles 30 sec. at 94°C, 40 sec. at 62°C and 40 sec. at 72°C, followed by a final extension step for 7 min. at 72°C. As *Mat* determinations by PCR were not conclusive, we eventually decided to use the Bo_1 strain as common parent in five pairings, which we expected to be no less than 40% successful due to the bipolar heterothallic mating system of *P*. *fijiensis* [16]. We, therefore prepared 15 ml of mycelium solution—as described above—of each parental strain and then mixed the sensitive *P*. *fijiensis* strain Bo_1 in a 1:1 ratio with each of the other aforementioned Costa Rican strains, hence in total five mixtures, which were incubated overnight at 27°C to recover from blending. The next day the inoculum mixtures were atomized on individual “Cavendish” banana plants, variety Grand Nain, using a spray device (#0267–6, Preval®, Chicago, USA) at both sides of the leaves until run-off. Each plant was six months old to ensure large leaves with more surface area for disease development. After inoculation, plants were maintained in the greenhouse for 14 weeks with the following growth regime: light (>300 μmol m^-2^ s^-1^) period of ~12 hours; day/night temperatures of 28°C/25°C with a relative humidity >90%. First necrosis and mature spots appeared around 65 days after inoculation (dai) and starting from that day, leaf pieces with the mature reproductive lesions were taken for ascospore discharge as described above. The first set of spores was observed and collected 73 days after inoculation from two crosses (Table 1) and 100 ascospores were isolated from each cross for further analyses.

**Table 1:** Crossing *Pseudocercospora fijiensis* strains^1^.

Cross	DMI sensitive parent 1	Propiconazole EC_50_ average score (mg.l^-1^)	DMI resistant parent 2	Propiconazole EC_50_ average score (mg.l^-1^)	Progeny
N1	Bo_1	0.020	CaM10_16	5.730	No progeny
N2			CaM10_6	11.750	Successful cross
N3			CaM7_19	5.125	No progeny
N4			CaM7_10	2.205	Incompatible cross^2^
N5			CaM10_21	6.349	Successful cross

^1^DMI sensitive strain (Bo_1), mating type *mat1-1*, was crossed to five *mat1-2* DMIs resistant strains (CaM10_16, CaM10_6, CaM7_19, CaM10_21) and one *mat1-1* resistant strain (CaM7_10). Crosses were performed directly after the preliminary sensitive assay to avoid possible loss of sexual fitness due to sub-cultivation.

^2^The *mat* gene configuration was unknown in the moment of the cross experiment.

### DArTseq marker generation

A set of 98 strains from each population was genotyped using DArTseq technology (www.diversityarrays.com). DNA samples were processed as described previously with few modifications [31]. The technology was optimized for *P*. *fijiensis* by using two restriction enzymes, i.e. *PstI* and *MseI*, rather than *PstI* only. The Restriction Enzyme (RE) overhangs differed from one another, allowing ligation of RE-site-specific adaptors. The *Pst*I-compatible adapter was designed to include Illumina flow cell attachment sequence, the sequencing primer sequence and a “staggered”, varying length barcode region, similar to the sequence reported by Elshire [32]. The reverse adapter contained the flow cell attachment region and a *Mse*I-compatible overhang sequence. Only “mixed fragments” (*Pst*I-*Mse*I) were effectively amplified by PCR. Equimolar amounts of amplification products from each sample of the 96-well microtiter plate were bulked and applied to c-Bot (Illumina) bridge PCR followed by sequencing on an Illumina Hiseq2000. Each generated marker had a sequence length of 68bp. Sequences generated from each lane were processed using proprietary DArT analytical pipelines [31]. In the primary pipeline the fastq files were processed to filter away poor quality sequences, applying more stringent selection criteria to the barcode region compared to the rest of the sequence. In that way the assignments of the sequences to specific samples carried in the “barcode split” steps were very reliable. Approximately 2,000,000 sequences per barcode/sample were identified and used in marker calling. Identical sequences were collapsed into “fastqcoll files” and a second pipeline was followed for further quality selection criteria as described [31]. Finally, the scored markers (presence/absence of restriction fragments) were represented in a 0/1 binary matrix to be used in the calculation of the genetic similarity (S1 Data).

### Genetic linkage maps

Approximately 5,400 DArTseq markers were generated for the segregating F_1_ populations N2 and N5 (Table 1 and S1 Data). As the DMI sensitivity trait showed a clear bimodal distribution (sensitive versus resistant), this trait was integrated as phenotypic marker in both genetic maps. The markers were filtered based on their co-segregation with the sensitivity trait and those with close linkage (<10cM) to the trait were selected for the construction of a linkage group to identify the genetic region of the responsible gene. The linkage group markers were sorted with the genetic mapping software JoinMap 4.1 [33] and, according to the positions of the recombination events, the strains were sorted to identify the chromosomal region harboring the sensitivity gene. Subsequently, the genetic map was linked to the physical map by aligning the DNA sequences of the DArTseq markers to the *P*. *fijiensis* CIRAD 86 reference genome version 2.0 (http://fungi.ensembl.org/Pseudocercospora_fijiensis_cirad86/Info/Index).

### *Pfcyp51* sequencing

The *Pfcyp51* genes of a total of 193 strains from both populations (98 strains from N2 and 95 strains from N5) were sequenced. To amplify the *Pfcyp51* gene, specific primers were used: *CYP51_Pfijien_F1* (5’-AAGGTCATATCGCAGG-3’) and *CYP51_Pfijien_R1* (5’-GAATGTTATCGTGTGACA-3’). A standard PCR mix was used in the following program; initiation with a 5 min. denaturation at 94°C followed by 35 cycles of 30 sec. denaturation at 94°C, 30 sec. annealing at 55°C and 90 sec. extension at 68°C with a final round of seven min. extension at 72°C. The DNA sequencing of the *Pfcyp51* amplicons was directly performed on the PCR products (Macrogen Europe, Amsterdam, The Netherlands). Full sequence coverage was obtained by using four primers: *CYP51_Mfijien_F2* (5’-ACAGAAACATCACCTCC-3’, *CYP51_Mfijien_F3* (5’-ATTGCTTCACTTTCATCC-3’), *CYP51_Mfijien_F4* (5’-CTCTACCACGATCTCGAC-3’) and *CYP51_Mfijien_R2* (5’-GATATGGATATAGTTGTC-3’). The obtained *Pfcyp51* sequences were assembled per strain in contigs (SeqMan application software Lasergene v8, DNASTAR^®^, Madison, USA) and aligned to gene model MYCFIDRAFT_30715 of the reference genome (*P*. *fijiensis* CIRAD 86, genome version 2.0, [11]), using the CLC Genomic Workbench software (version 7.5.2, CLC Bio-Qiagen, Aarhus, Denmark).

## Results

### Progeny generation and DMI segregation

Successful crosses were accomplished after two experimental failures where we empirically determined the critical number of sub-cultivations of the parental strains, which should not be more than two in order to maintain sexual fitness. Among the five evaluated crosses, four combinations produced mature lesions at 65 dai, but ascospores were only discharged from N2 (Bo_1 x CaM10_6) and N5 (Bo_1 x CaM10_21) at 73 dai (Table 1). Other crosses failed, apparently due to identical *mat* genotypes.

**Fig 1:**
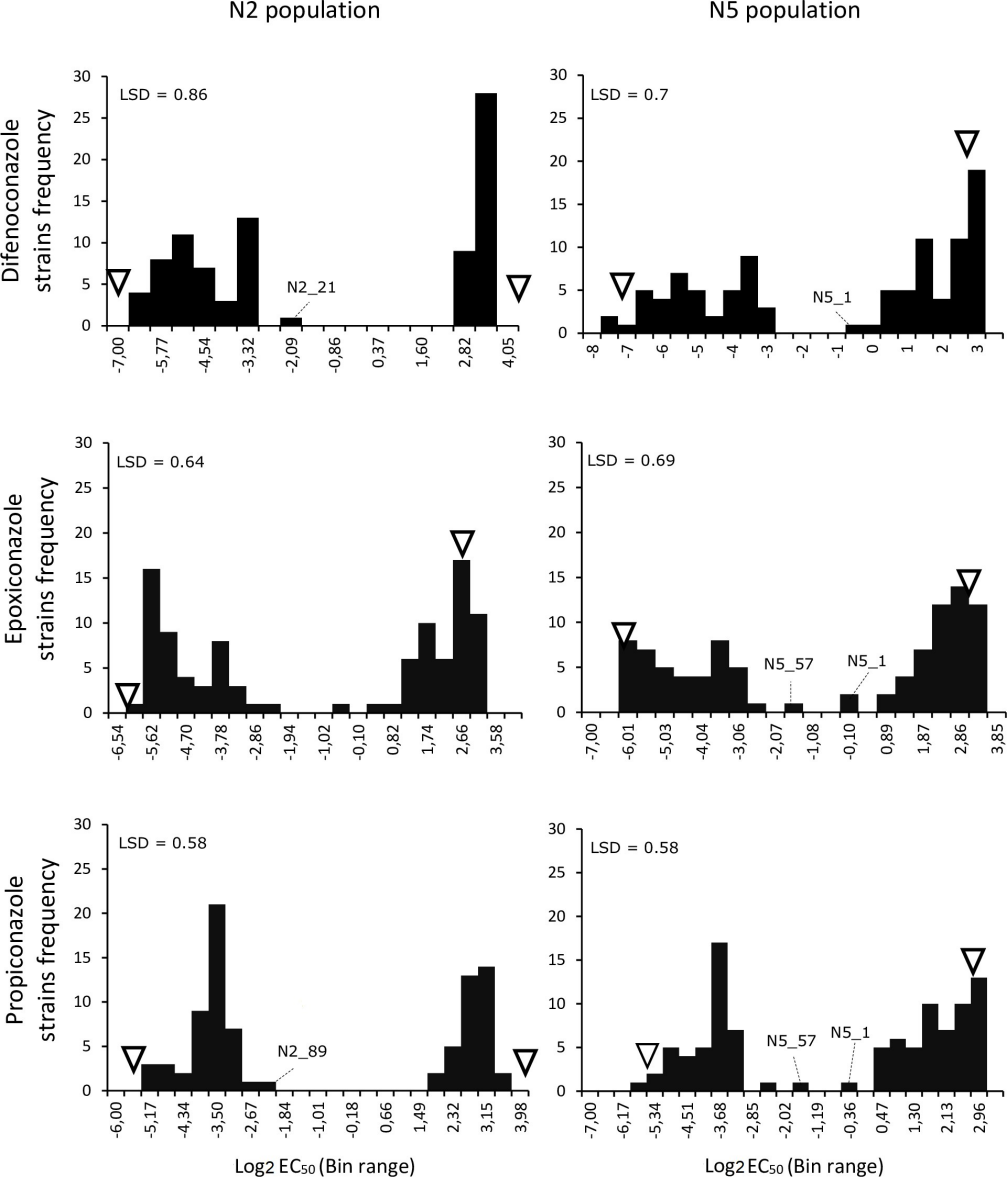
Segregation of DMI sensitivity in the *Pseudocercospora fijiensis* mapping population N2 and N5. Histograms of the log_2_ average EC_50_ data per fungicide are shown for each population. Resistant and sensitive strains show the same sensitivity response to all three DMI fungicides respectively. A minority of the progeny strains showed intermediate phenotypes in some fungicides (EC_50_ thresholds between resistant >1 mg.l^-1^, Intermediates from 0.2–1 mg.l^-1^ and sensitive ≤0.2 mg.l^-1^).The Bin range was based on the lower and upper intervals of the standard error of the difference of the log_2_ means. The EC_50_ positions of the parental strains are marked with triangles. Least Significant Difference (LSD) values are shown above the histograms. Positions of strains with intermediate response are shown with dash lines.

A total of 200 progeny strains was characterized for DMI sensitivity using epoxiconazole, propiconazole and difenoconazole (Table 2). The segregation ratios for sensitivity versus resistance of the N2 and N5 progenies were 47:53 and 44:56, respectively, according to an expected 1:1 ratio for a single gene inheritance. Hence, examination of the sensitivity response in both F_1_ populations revealed a clear bimodal distribution (Fig 1, S1 and S2 Figs). Despite these bimodal distributions, four strains (N2_21, N2_89, N5_1, and N5_57) had EC_50_ values out of the selected thresholds range for sensitive or resistant groups and were regarded as intermediate response to the tested DMIs (Fig 1). These four strains were included in the mapping generation. Based on the sequence analyses, only N5_1 was regarded as resistant, whereas the others were considered sensitive. The average EC_50_ scores for each strain are shown in the S2 Table.

**Table 2:** Summary of the DMIs EC_50_ data for the two *Pseudocercospora fijiensis* mapping populations N2 and N5^1^.

Populations		Difenoconazole					Epoxiconazole					Propiconazole				
Code	Trait^2^	Max	Min	Av (SD)	%	RF	Max	Min	Av (SD)	%	RF	Max	Min	Av (SD)	%	RF
**N2**	**S**	0.213^2^	0.009	0.05 (0.04)	47	-	0.19	0.015	0.04 (0.04)	47	-	0.21^2^	0.027	0.07 (0.03)	47	-
	**R**	>10.24	5.669	8.82 (1.268)	53	187.57	>10.24	1.351	4.78 (2.08)	53	108.65	>10.24	3.732	7.91 (2.1)	53	106.90
**N5**	**S**	0.108	0.005	0.04 (0.03)	44	-	0.315^2^	0.011	0.05 (0.05)	44	-	0.273^2^	0.015	0.07 (0.04)	44	-
	**R**	7.664	0.597^2^	4.30 (2.21)	56	107.43	9.459	0.748^2^	5.30 (2.22)	56	98.47	7.233	0.683^2^	3.41 (1.94)	56	94.22

^1^Indicated are the highest (Max) and lowest (Min) values that were obtained in the discretely segregating sensitive or resistant groups as well as their average values (Av) Standard deviation (SD), the percentage of strains in each category (%) and the average resistance factor (RF) of the resistant segregants. ^2^Sensitivity trait: S = sensitive, R = resistant.

^2^Four strains showed intermediate phenotypes.

### Genetic linkage maps

**Fig 2:**
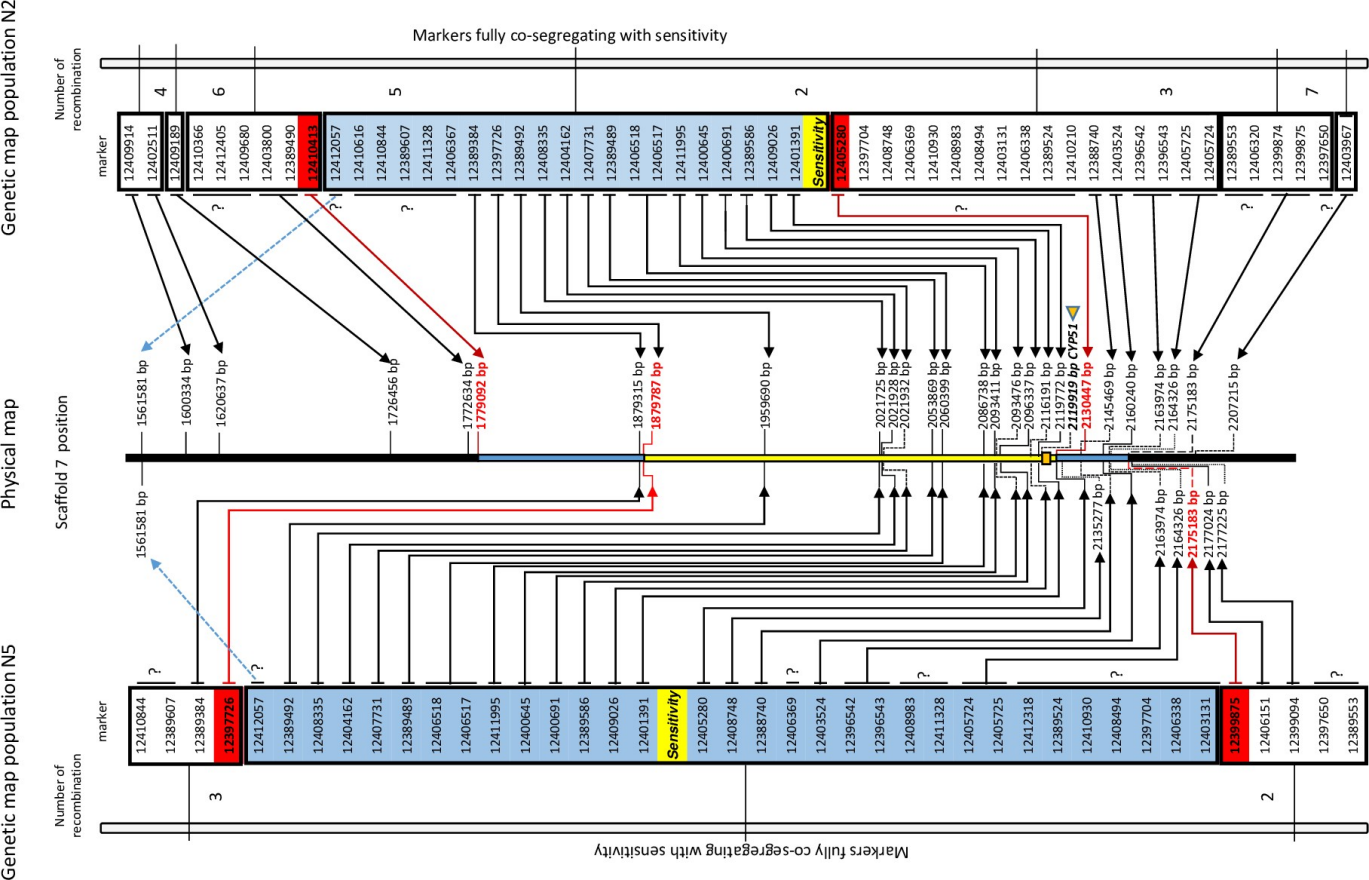
Integration of the *Pseudocercospora fijiensis* N2 (left) and N5 (right) genetic linkage maps with the physical map (Partial Scaffold_7) of the genomic reference *P*. *fijiensis* CIRAD86 (middle; *Mycosphaerella fijiensis* version 2.0).

We used the DArTseq markers to construct two linkage maps. From population N2, 53 markers were selected with 17 markers in coupling phase to resistance and 36 markers in coupling phase to sensitivity to DMIs (S4 Fig and S1 Table). The markers were clustered in seven groups based on their segregation patterns. Thirty-three of the markers were placed onto scaffold 7 of the physical map of *P*. *fijiensis* (S1 Table). The recombination events for the seven groups in the N2 population are shown in the right panel of Fig 2. From 53 markers, 21 fully co-segregated with sensitivity to the DMIs. Markers 12410413 and 12405280 were identified as the flanking markers of the sensitivity trait with physical positions scaffold_7:1,779,092 bp and scaffold_7:2,130,447 bp, respectively, and a physical distance of 351,355 bp.

The genetic map was generated using DArTseq markers. The sensitive trait is taken as phenotypic marker in the genetic map (marker in yellow). The number of recombinations between the markers is indicated between the linkage groups. Markers perfectly co-segregating with the sensitivity trait are indicated in blue, with the direct flanking markers depicted in red for both populations. The area of the co-segregating markers for each population is presented as a light blue line and the overlapping region is depicted in yellow on the physical map. The genetic and the physical map are linked for each marker by arrows. The flanking markers are indicated in red while the remaining markers are printed in black. Genetically linked markers without a position in the physical region are indicated with a question mark (?) and unpositioned markers are placed with a light blue dashed line. The position of *Pfcyp51* is marked in bold letters with a yellow triangle and is represented by a yellow box. From population N5, 41 markers were selected with eight markers in coupling phase with resistance and 33 markers in coupling phase with sensitivity to the tested DMIs (S4 Fig and S1 Table). The markers were clustered in three groups by their genetic distance, 27 of were on scaffold 7. All recombination events for the N5 population are shown in the left panel of Fig 2. From the 41 markers, 32 fully co-segregate with sensitivity to the tested DMIs. Markers 12397726 and 12399875 were identified as the flanking markers with positions scaffold_7:1,879,787 bp and scaffold_7:2,175,183 bp, respectively, and a physical distance of 295,396 bp.

The order of the genetic markers in both the N2 and N5 populations was in full agreement (Fig 2). The N2 and N5 populations share 38 markers and 17 markers from both maps show inconsistencies or low coverage scores, which were therefore omitted and not placed in the reference physical map. Markers with inconsistences between the physical and the genetic maps are markers 12412057 on scaffold_7—but in a displace position—12,412,405 on scaffold_27, marker 12397704 on scaffold_6 and marker 12410210 on scaffold_5. Since they co-segregated with the groups of markers close to or in the area carrying the sensitivity locus, we assume there are either differences between the sequences of the CIRAD86 reference genome and the parental/progeny strains or there are a few errors in their positioning on the physical map. The positions of all markers are indicated in Fig 2 and a summary of the information is compiled in S1 Table and S4 Fig. Based on the flanking markers, there is an overlapping region for the N2 and N5 populations of 250,660 bp between scaffold_7:1,879,787 (marker 12397726) and scaffold_7:2,130,447 (marker 12405280). This genetic window harbours 53 putative genes among which is *Pfcyp51*, located at scaffold_7:2,119,919–2,121,685 (Fig 2, S5 Fig and S3 Table).

### Molecular analyses of the *Pfcyp51* configuration in the N2 and N5 progenies

**Fig 3:**
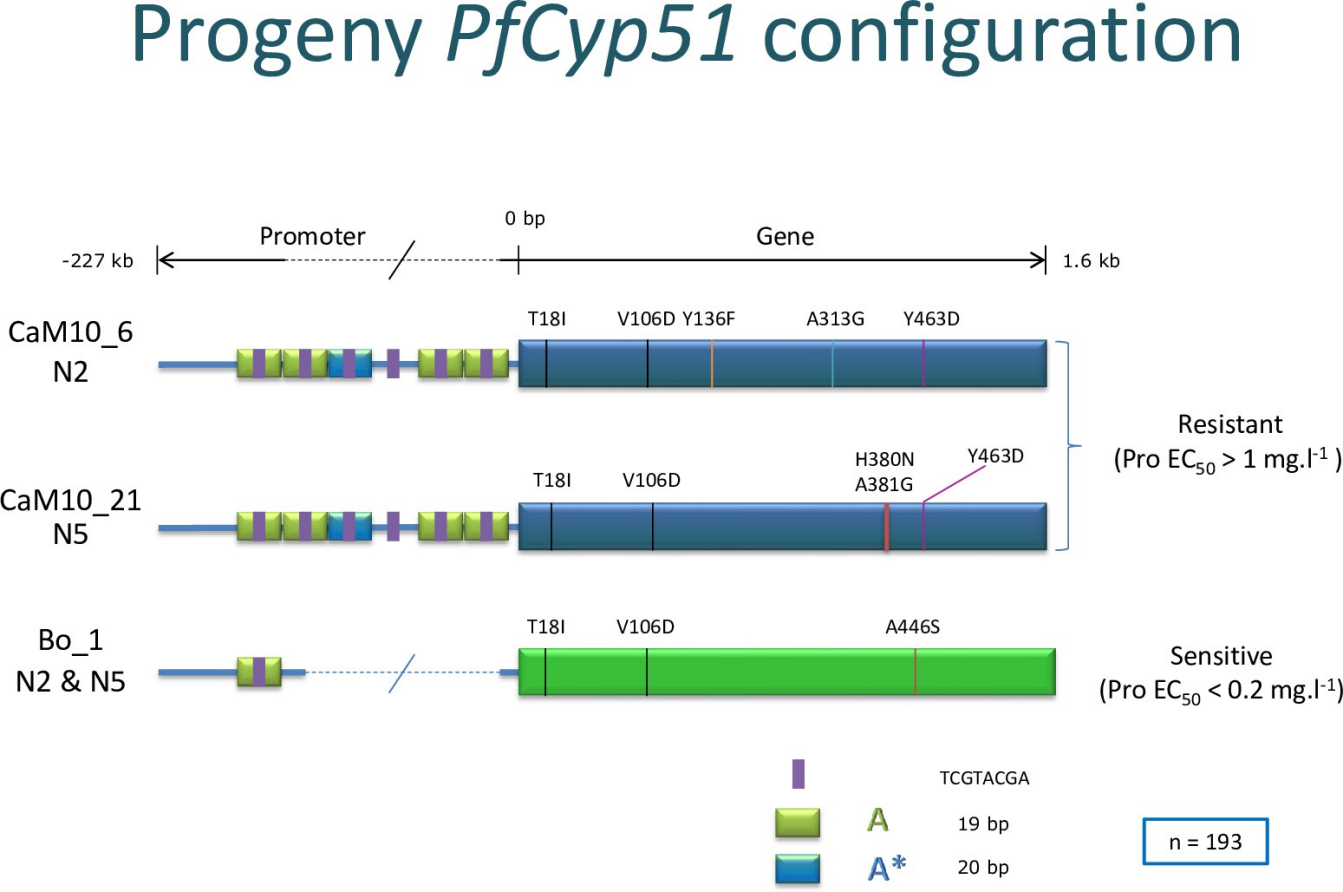
Schematic visualization of the *Pfcyp51* gene configuration derived from the *Pseudocercospora fijiensis* crossing partners and segregating progenies, based on the expressed phenotypes towards three DMI fungicides. All progeny strains exclusively showed parental genotypes. Resistant strains have promoter insertions while sensitive strains have no insertions. Mutations in the coding domain are marked with colored lines and the resulting aa substitutions.

Analysis of the *Pfcyp51* gene, including the promoter, revealed that all *P*. *fijiensis* progeny strains only had parental genotypes (S2 and S3 Data). Resistant strains carried the *Pfcyp51* gene encoding protein modifications T18I and V106D, which have no DMI phenotypic consequences, and three other substitutions related to DMI resistant described in Fig 3, whereas all sensitive strains were identical to the wild type genotype of the parental strain Bo_1, lacking any insertion in the promoter region and a *Pfcyp51* sequence encoding the non-phenotypical T18I, V106D and A446S amino acid (aa) modifications compared with the reference sequence of *P*. *fijiensis* CIRAD86 (S2 Table). However, all resistant progenies from either population contained a 103 bp promoter insertion located 94 bp upstream of the reference *Pfcyp51* start codon. The insertion is accompanied with an 18 bp substitution “GGACCACTCGAACATCAC”. (reference position MYCFIscaffold_7:2121783, *Mycosphaerella fijiensis* v2.0, JGI) and is composed of repeated elements interspersed with non-repeated sequences. The repeated element is described in [10, 15] and possesses a palindromic core. In total, three exact copies and a single modified copy of this element–from here identified as element A—are present in the insertion. The modified copy (A*) carries an additional one bp substitution, resulting in the sequence “T**A**AAATCTCGTACGATAGCA. All sensitive strains have only one A element in their promoter [10] (Fig 3). So, when taking into account this single A element in wt Bo_1, resistant progeny strains contained five A copies (Fig 3 and S3 Fig). All resistant progeny strains from the N2 population share *Pfcyp51* substitutions resulting in the aa modifications Y136F, A313G and Y463D originating from their resistant parent CaM10_6 (Fig 3 and S2 Table). The resistant progeny from the N5 population have substitutions resulting in the aa changes H380N, A381G and Y463D, originating from the parental resistant strain CaM10_21 (Fig 3 and S2 Table). All *Pfcyp51* sequences from parents and progenies of both the N2 and N5 populations showed a perfect match with the segregating phenotypes (sensitive: resistant = 1:1, S2 Table). From the aforementioned four strains with intermediate behavior, strains N2_21, N2_89 and N5_57 contained the *Pfcyp51* sequence of the sensitive parent and strain N5_1 had the configuration of the resistant parent. Hence, their intermediate phenotypes were due either to measurement errors or caused by other genomic modifications.

## Discussion

Understanding fungicide resistance phenomena is crucial for banana crop sustainability as well as human and environmental health. For this reason, we decided to perform an unbiased genetic analysis to identify any other underlying factors for the resistance to DMIs in *P*. *fijiensis*. Therefore, we generated two new high marker density genetic linkage maps after crossing sensitive and resistant *P*. *fijiensis* strains. These crosses were not at all routine, required a pragmatic approach compared to previous reports [34], and eventually resulted in two mapping populations. One of the most challenging tasks was to perform the *P*. *fijiensis* crosses. First we were not able to unequivocally determine the mating type of each strain, which previously also appeared difficult [30], despite the fact that the *P*. *fijiensis mat* genes had earlier been cloned [16]. Our pragmatic approach is similar to the protocol being used for the related dothideomycete wheat pathogen *Zymoseptoria tritici* [35–37]. Recent genome data show that sub-culturing fungal strains frequently results in chromosome loss [38, 39], and hence it is conceivable that our failures to successfully cross *P*. *fijiensis* was related to the number of sub-cultivations of each candidate parent [40]. The moment we reduced sub-cultivations to maximally two, crosses proved to be successful, resulting in viable and sufficient progeny strains for formal genetic analyses. Hence, we conclude that subsequent sub-cultivation steps affect sexual fitness, although we do not know whether this has to do with the loss of essential chromosomes. Alteration of fungal properties by sub-cultivation not only affects mating but also pathogenicity as observed by Krokene and Solheim in the blue-stain fungus *Ceratocystis polonica* [41] and by Kashino et al. in *Paracoccidioides brasiliensis* [42]. Krokene and Solheim also observed alterations in the ability to grow under oxygen-deficient and reduction in growth rate in *C*. *polonica* [41].

Despite the bimodal distribution into sensitive and resistant progeny, the wide range of EC_50_ values’ in each group was substantial and awaits further explanation. Potentially, other individual factors such as minor fungicide resistance genes, genes related to stress responses or growth rates are involved. Nonetheless, the DMI response difference between sensitive and resistant strains was clear and separates them into two major and discrete groups with an approximate differential resistance factor of 100 (Table 2), resulting in a 1:1 segregation. This phenotypic segregation was in accordance with the segregation of the *Pfcyp51* gene. The progeny was no different when compared to the parents, despite the quantitative expression of DMI sensitivity [5, 12, 15]. None of the sensitive strains contained amino acid substitutions or insertions in the promoter region of *Pfcyp51*, which were previously correlated with reduced efficacy in DMIs [5, 10]. Furthermore, based on the genetics maps, no evidence was observed for the presence of any other sensitive genetic region in either progeny. This strongly supports the presence of the *Pfcyp51* gene as the single explanatory factor for DMI sensitivity. In only four progenies strains an alternative explanation seems appropriate but was not apparent from the generated data set and might equally be due to experimental error.

The genetic window explaining DMI sensitivity contained 53 genes, of which the majority lack any functional clue. Predicted gene Id96804 encodes a putative transcription factor, which might regulate expression of (minor) genes that contribute to DMI resistance and predicted gene Id86816 encodes a putative transporter that might facilitate increased efflux [43, 44] (S3 Table). However, the overruling factor seems to be *Pfcyp51* that also maps to this exact region (Fig 2 and S4 Fig) and which accords with its importance in other–related–fungi [14] as well as with the evidence of *Pfcyp51* overexpression that recently became available [5, 8, 10, 15]. Therefore, despite the presence of other genes in the mapped genomic region, we propose that modifications of the *Pfcyp51* gene and its promoter are the driving molecular force for DMI fungicide resistance.

Common mechanisms described for the loss of sensitivity to DMIs are increased efflux of the fungicides from the cells [14, 45], adaptation and overexpression of the fungicide target gene [9, 10, 22, 46] and cellular alterations that reduce the toxicity of the fungicides, such as modulation of the targeted biosynthesis pathway [13]. Recently, we showed that modulation of the promoter and coding domain of *Pfcyp51* explains reduced DMI sensitivity [10]. Other fungal species, such as *Candida albicans* and *Aspergillus graminearum*, accommodate more than one of these mechanisms [13, 20, 47]. Our data suggests that in *P*. *fijiensis* modification and consistent alteration in the promoter region of the *Pfcyp51* are the most plausible causes of the reduced sensitivity to DMIs. The current study underscores these observations as our unbiased linkage approach did not map any additional contributing factor to DMI sensitivity.

In comparison with the DMI resistance mechanisms present in other fungi [14, 20] it is remarkable that we have exclusively identified a single role for *Pfcyp51* in DMI sensitivity. The lack of alternative quantitative mechanisms might indicate that we are just at the beginning of *P*. *fijiensis* DMI resistance development, illustrative for the recent development in DMI resistance in Latin America. Common DMI resistance mechanisms in other fungi include increased fungicide efflux or alternative ergosterol synthesis pathways [13, 14] which may therefore become more common in the future if the fitness penalty for these mechanisms is sufficiently low [13, 14]. However, this largely depends on future application of DMI fungicides. The current situation on reduced DMI efficacy has already resulted in a fallback strategy avoiding DMIs and increased use of protectants and mineral oil [1].

Interestingly, the overall sensitivity loss in population N2 was higher than in N5. Based on the similarity of the promoter configuration we assume that the expression of the gene is comparable (Table 2). Therefore, the sensitivity difference might be explained by the non-synonymous mutations present in the *Pfcyp51* gene (Fig 2). Resistant progeny from N2 harbour aa substitutions Y136F, A313G and Y463D, while those in N5 comprise H380N, A381G and Y463D. The differentiating substitutions at positions 136 and 313 are particularly important since these are located in the substrate binding site [5, 15].

Unlike other single site fungicide interactions, CYP51 substitutions often affect individual, or a subset of DMIs compounds, with generally incomplete cross-resistance across the whole class [14]. This particular mode of interaction of the CYP51 protein can explain the unusual behaviour and the steep increase of DMI resistance in the banana plantations. It is likely that the quantitative resistance response–as observed for DMI resistance in the field—is due to an accumulation of different *Pfcyp51* non-synonymous mutations in response to the apparent selection pressure, which also and alternatively can be due to a dramatic synergistic and epistatic effect explained by *Pfcyp51* overexpression under high selection pressure [10].

Since there was no recombination events close to the *Pfcyp51* gene, we also conclude that sexual reproduction apparently does not contribute to the important *Pfcyp51* promoter modulations, which therefore awaits further mechanistic explanations. Although, our data have not shown any modulating effect of sexual reproduction on *Pfcyp51*, the versatility of the fungus through an almost continuous production of offspring throughout the year is undeniable. Current spraying practices likely significantly contributed to the accumulation and actually fixation of strobilurin resistance, as was recently explained in *Z*. *tritici* [48]. This should—evidently—be a huge concern for the banana industry since the maximum number of fungicide applications seems to have plateaued and thus the efficacy of the treatments could be on the verge of breaking. In other species there are few indications that a temporal suspension of DMI applications results in a subsequent decrease of resistant strains due to fitness penalty and stability restrains in the *Pfcyp51* gene [23, 26, 49], although in a limited number of cases this strategy was shown to be functional [13, 50]. Resistance breeding, but also biological control measures and the development of alternative non-DMI fungicides are considered as the most promising options for a more balanced and hence sustainable black Sigatoka management.

## Supporting information

S1 Fig Plots of the calculated EC_50_ values and residuals of the interaction of the *Pseudocercospora fijiensis* segregating N2 population with the three DMI fungicides. A) difenoconazole, B) epoxiconazole and C) propiconazole.
(TIF)

S2 Fig Plots of the calculated EC_50_ values and residuals of the interaction of the *Pseudocercospora fijiensis* segregating N5 population with the three DMI fungicides. A) difenoconazole, B) epoxiconazole and C) propiconazole.
(TIF)

S3 Fig Analysis of the insertions in the promoter region of *Pfcyp51* gene in *Pseudocercospora fijiensis* progeny strains in both the N2 and N5 mapping populations. The promoter modifications start at -94 bp upstream of the *Pfcyp51* start codon of the reference sequence. Element “A” is shown in blue boxes together with the arrangement of the palindromic sequence TCGTACGA shown in green boxes. Element “A*” is shown in red as a partial construction of element “A” in purple. Negative values in the right bottom represent the positions from the beginning of the insertion related to the start codon of the gene.
(TIF)

S4 Fig *Pseudocercospora fijiensis* strains sorted according to the positions of the recombination events in the chromosomal region harbouring the *Pfcyp51* sensitivity gene. A) Mapping population N2 and B) Mapping population N5. The markers descending from the highly sensitive parent are coded “a” and shown in light red boxes, whereas the chromosomal segments inherited from the resistant parent genotype are coded “b” and shown in light green boxes. The unknown values are represented by dashes in grey boxes (-). The DArTseq markers fully co-segregating with sensitivity are shown in light blue with the sensitivity trait shown in yellow. The flanking markers are shown in red.
(TIF)

S5 Fig Scheme of the position of the 53 putative genes found in the overlapping genetic window of the *Pseudocercospora fijiensis* N2 and N5 mapping populations that contains the sensitivity region. The *cyp51* gene is highlighted in green. The figure was based on the assembly found in Ensemble fungi portal website, *Pseudocercospora* fijiensis CIRAD 86 (CGA_000340215) (Mycfi2), location: KB446561: 1,885,523–1,935,524. Information at: http://fungi.ensembl.org/Pseudocercospora_fijiensis_cirad86_gca_000340215/Location/View?db=;g=MYCFIDRAFT_30715;r=KB446561:1881497-2132157;t=EME80226.
(TIF)

S1 Table Sequence information of the DArTseq markers used to generate the *Pseudocercospora fijiensis* N2 and N5 population linkage maps^1^. ^1^The markers were selected based on their co-segregation with the sensitivity trait.
(DOCX)

S2 Table Sequence information of the *Pfcyp51* gene of the *Pseudocercospora fijiensis* parental strains and the N2 and N5 progenies and their average EC_50_ scores against the three DMI fungicides difenoconazole, epoxiconazole and propiconazole. *Strains with scores <0.2 mg.l^-1^ were classified as sensitive and strains with scores >1.00 mg.l^-1^ were classified as resistant. Strains with question marks (?) remained undetermined.
(DOCX)

S3 Table Information of the 53 putative genes found in the overlapping genetic window of the *Pseudocercospora fijiensis* N2 and N5 mapping populations that contains the sensitivity region^1^. ^1^The information of the genes was collected from the Joint Genome Institute, *Mycosphaerella fijiensis (Pseudocercospora fijiensis)* v2.0 portal. https://mycocosm.jgi.doe.gov/pages/search-for-genes.jsf?organism=Mycfi2.
(DOCX)

S1 Data DArTseq marker generation base on parental strains and N2 and N5 populations. (XLSX)

S2 Data Alignment of the *Pfcyp51* gene of the *Pseudocercospora fijiensis* parental strains and the N2 progeny. (FAS)

S3 Data Alignment of the *Pfcyp51* gene of the *Pseudocercospora fijiensis* parental strains and the N5 progeny. (FAS)
