# Supplementary figures and images for: Pfcyp51 exclusively determines reduced sensitivity to 14α-demethylase inhibitor fungicides in the banana black Sigatoka pathogen Pseudocercospora fijiensis
Source: PLoS One. 2019 Oct 17;14(10):e0223858. doi: 10.1371/journal.pone.0223858 (PMC6797121; doi:10.1371/journal.pone.0223858)

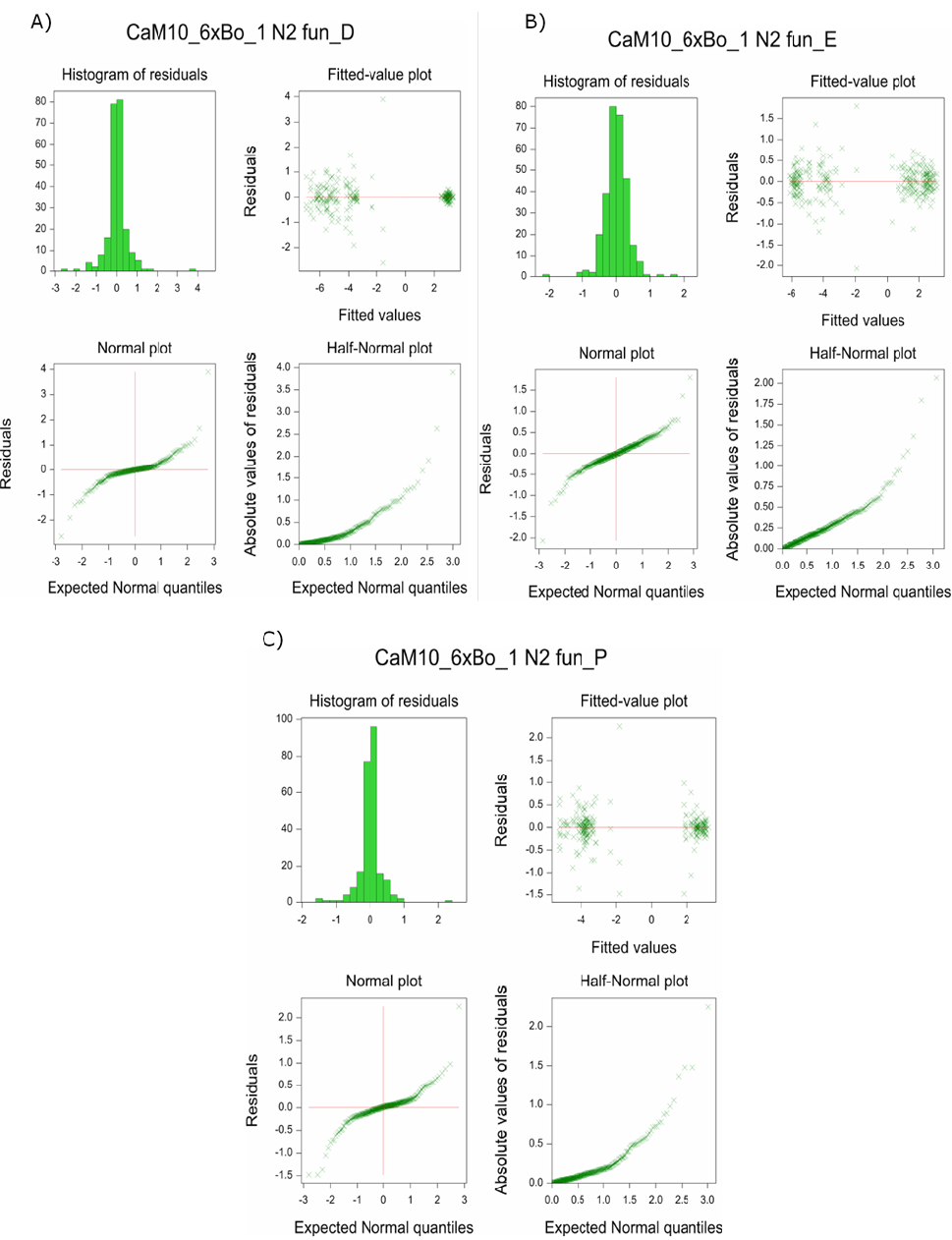

Supplement: S1 Fig — A) difenoconazole, B) epoxiconazole and C) propiconazole. (TIF) [file pone.0223858.s001.tif]

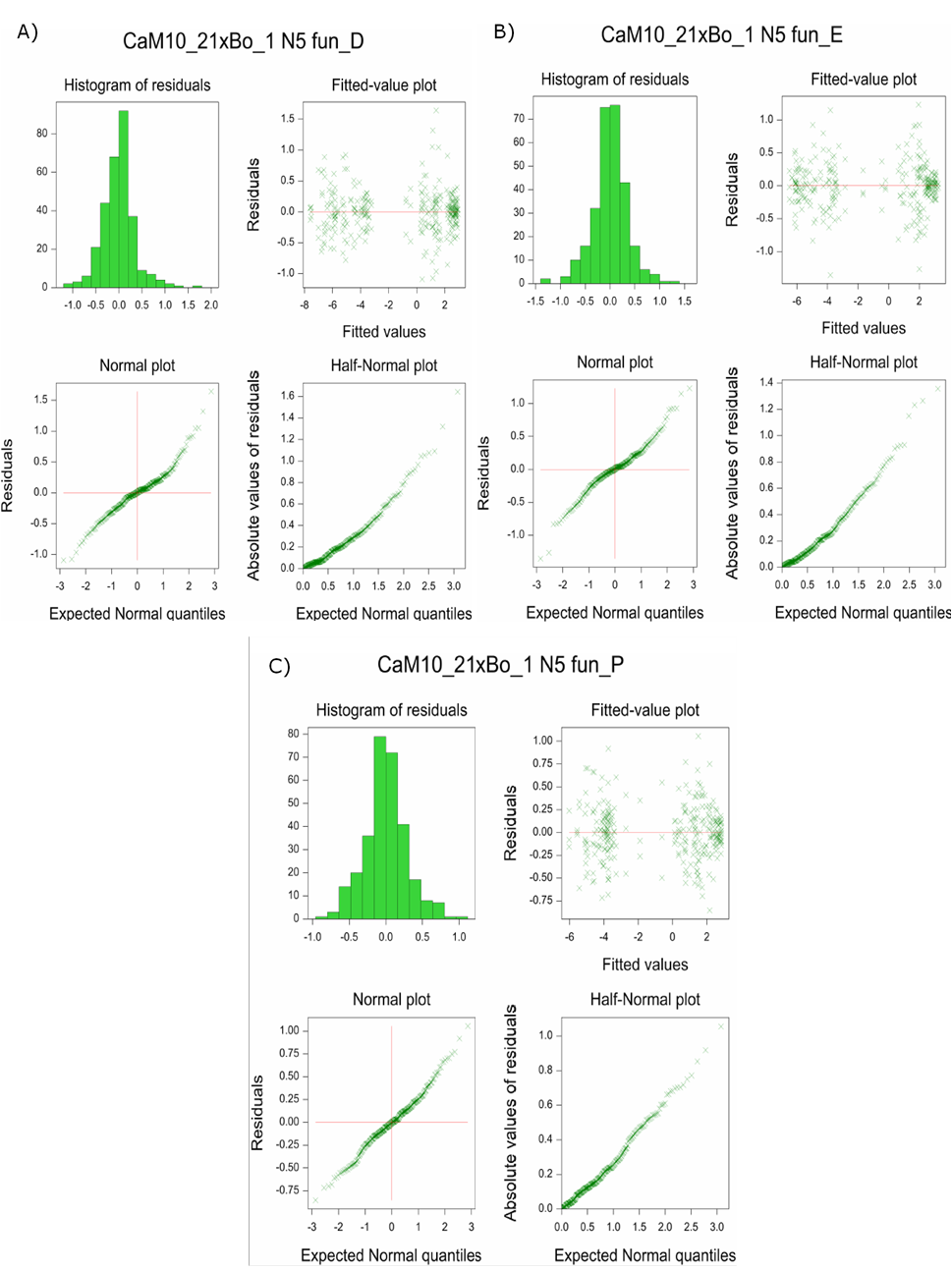

Supplement: S2 Fig — A) difenoconazole, B) epoxiconazole and C) propiconazole. (TIF) [file pone.0223858.s002.tif]

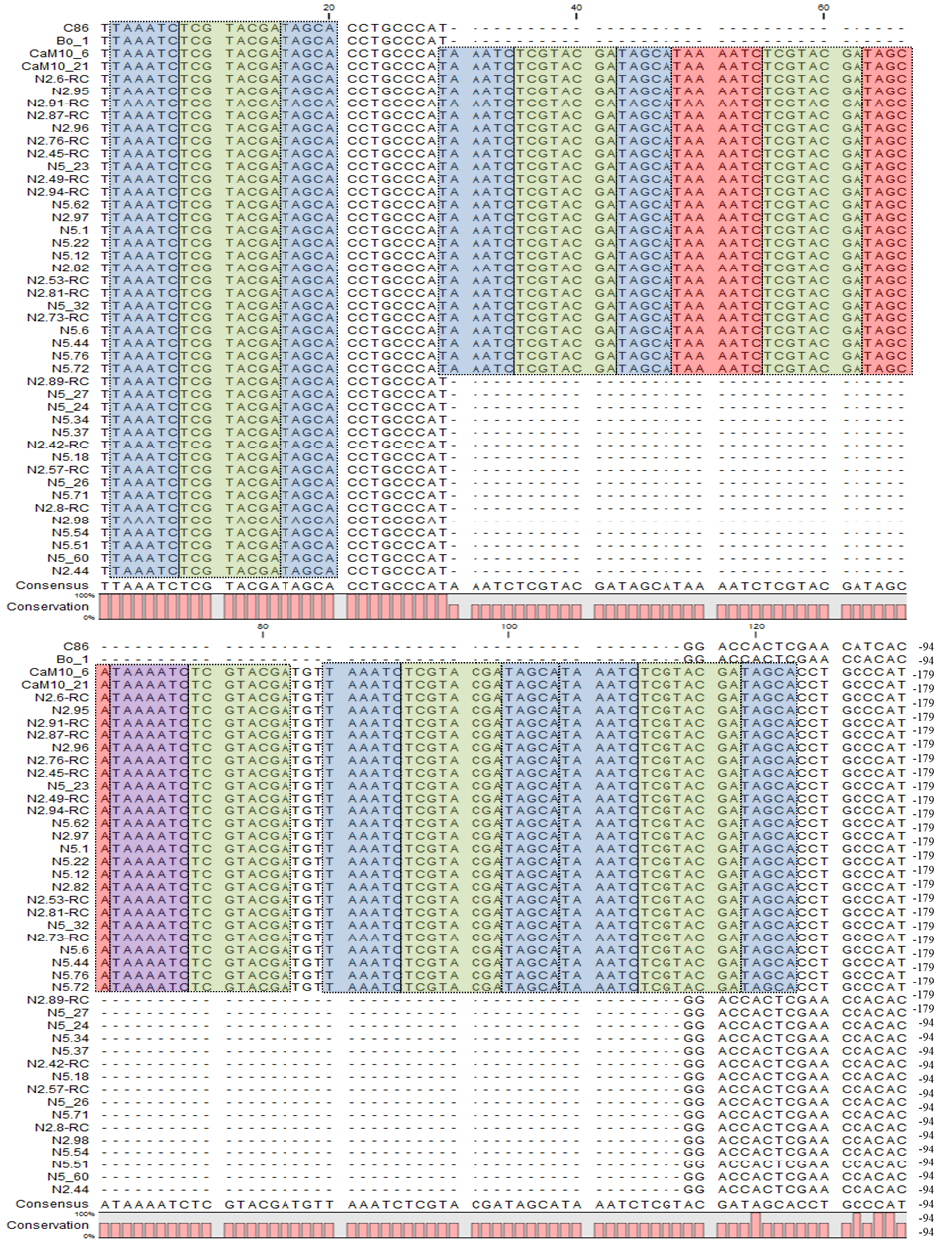

Supplement: S3 Fig — The promoter modifications start at -94 bp upstream of the Pfcyp51 start codon of the reference sequence. Element “A” is shown in blue boxes together with the arrangement of the palindromic sequence TCGTACGA shown in green boxes. Element “A*” is shown in red as a partial construction of element “A” in purple. Negative values in the right bottom represent the positions from the beginning of the insertion related to the start codon of the gene. (TIF) [file pone.0223858.s003.tif]

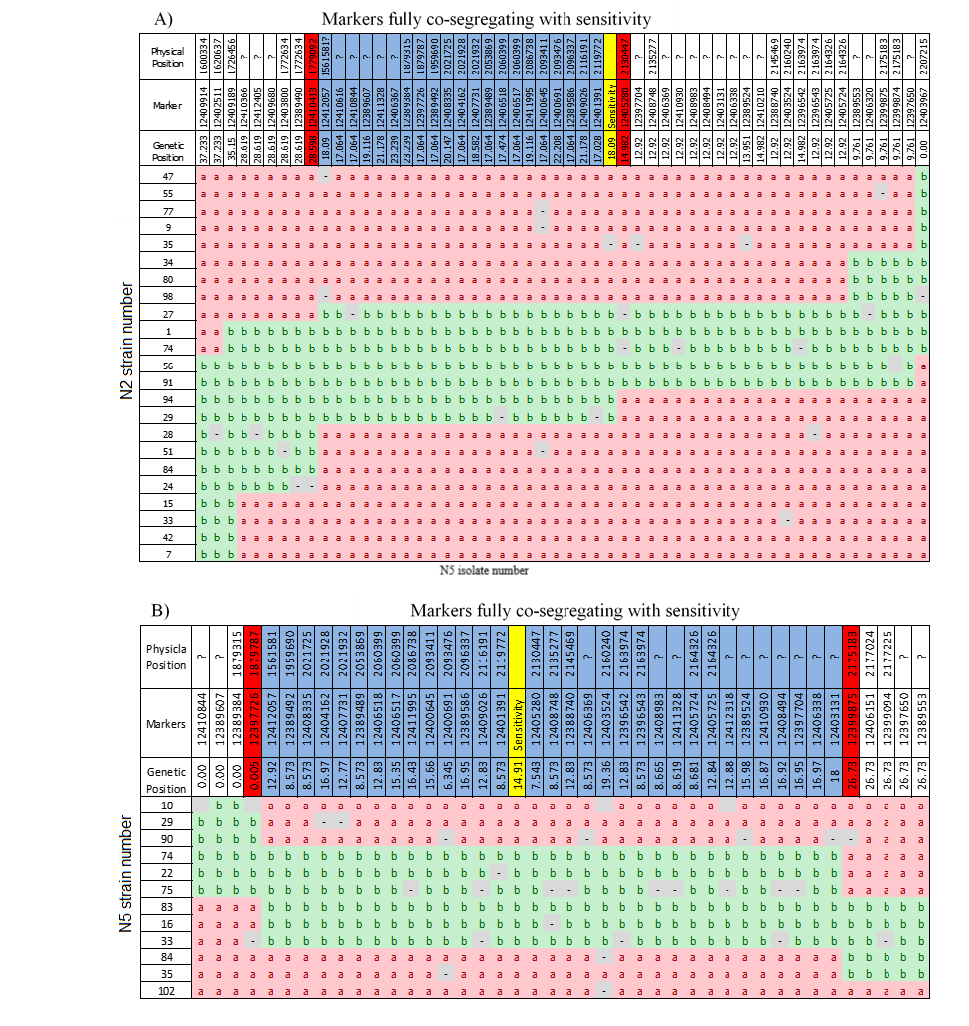

Supplement: S4 Fig — A) Mapping population N2 and B) Mapping population N5. The markers descending from the highly sensitive parent are coded “a” and shown in light red boxes, whereas the chromosomal segments inherited from the resistant parent genotype are coded “b” and shown in light green boxes. The unknown values are represented by dashes in grey boxes (-). The DArTseq markers fully co-segregating with sensitivity are shown in light blue with the sensitivity trait shown in yellow. The flanking markers are shown in red. (TIF) [file pone.0223858.s004.tif]

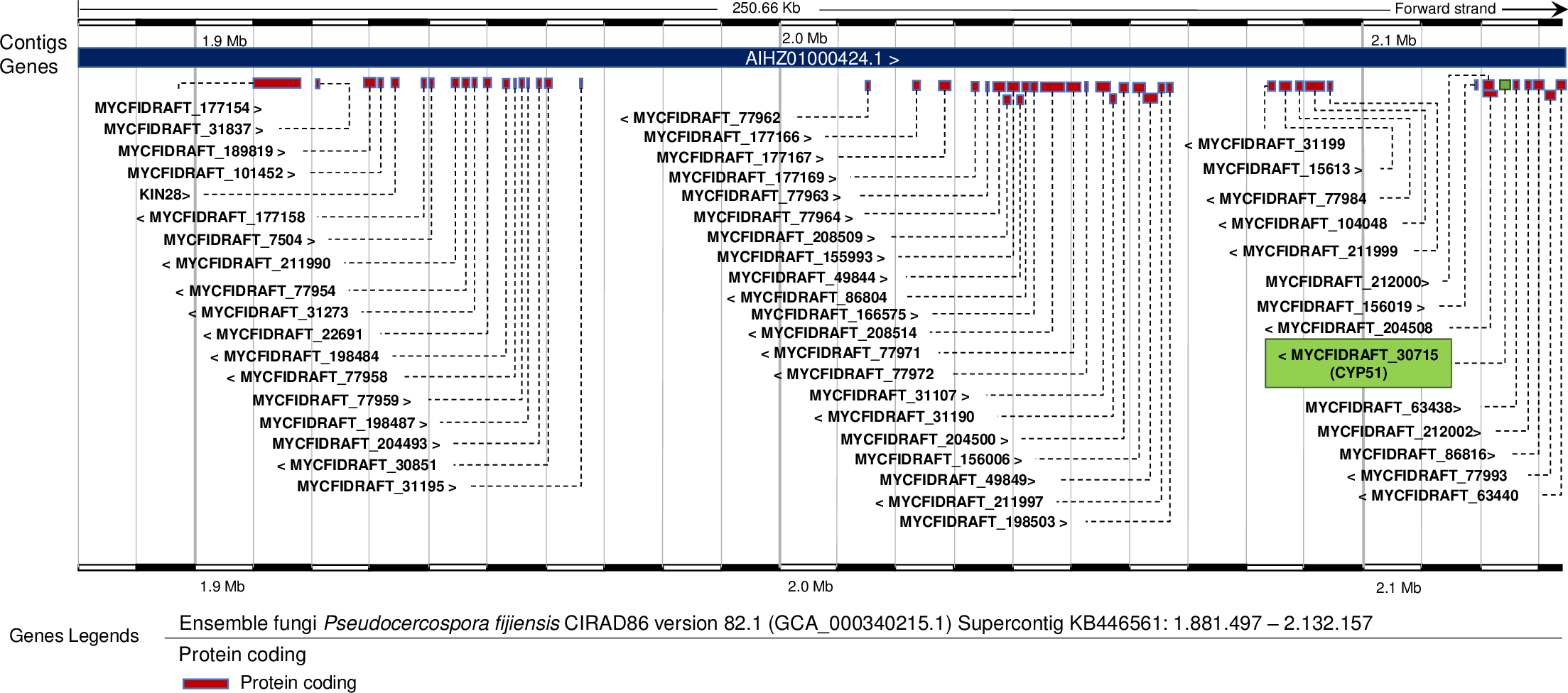

Supplement: S5 Fig — The cyp51 gene is highlighted in green. The figure was based on the assembly found in Ensemble fungi portal website, Pseudocercospora fijiensis CIRAD 86 (CGA_000340215) (Mycfi2), location: KB446561: 1,885,523–1,935,524. Information at: http://fungi.ensembl.org/Pseudocercospora_fijiensis_cirad86_gca_000340215/Location/View?db=;g=MYCFIDRAFT_30715;r=KB446561:1881497-2132157;t=EME80226. (TIF) [file pone.0223858.s005.tif]
