# Supplementary material for: Pfcyp51 exclusively determines reduced sensitivity to 14α-demethylase inhibitor fungicides in the banana black Sigatoka pathogen Pseudocercospora fijiensis
Source: PLoS One. 2019 Oct 17;14(10):e0223858. doi: 10.1371/journal.pone.0223858 (PMC6797121; doi:10.1371/journal.pone.0223858)
Supplement: S1 Table — 1The markers were selected based on their co-segregation with the sensitivity trait. (DOCX) [file pone.0223858.s006.docx]

| **N2** | **N5** | **Genome Reference %ID** | **coverage score** | **Coverage %** | **Scaffold best hit** | **Reference Genome Position** | | **Marker Sequence** | **N2 sensitivity segregation score** | | **N5 sensitivity segregation score** | |
| --- | --- | --- | --- | --- | --- | --- | --- | --- | --- | --- | --- | --- |
| **Marker ID code** | |  |  |  |  | **Beginning** | **End** |  | **Sensitive** | **Resistant** | **Sensitive** | **Resistant** |
| 12409914 | - | 100 | 68.00 | 98.55 | 7 | 1600334 | 1600402 | TGCAGATTTATGATGGGTGGCTCATTCGGCCTTTG  GTTGGGAAGCAGATTGAGCAGCGAGAGCGAGTTT | 0.195 | 0.918 | - | - |
| 12402511 | - | 100 | 68.00 | 98.55 | 7 | 1620637 | 1620705 | TGCAGTTCGACTTCTTCGAGTTCAATGCCATCTGT  GCTGCGAATGCGCCAGTTCTCCTTCCGCTTGGCA | 0.167 | 0.913 | - | - |
| 12409189 | - | 100 | 35.00 | 50.72 | 7 | 1726456 | 1726491 | TGCAGCTGGAGAGGAAGTCTGGTCGAACCATTCTT  ACAGATCGGAAGAGCGGTTCAGCAGGAATGCCGA | 0.205 | 0.940 | - | - |
| 12410366 | - | many hits | ? | ? | ? | ? | ? | TGCAGTAGCAAATTTGCTACTGCCTAGGCGCCCTA  AGGAGTACTCTAATCGATGCTAGACGGGACTAGT | 0.091 | 0.960 | - | - |
| 12412405 | - | 100 | 65.00 | 94.20 | 27(?) | 6204 | 6269 | TGCAGCCTATAGAATTCGTATACAACAATAGTGT  ATACTTATCTACTAAGGTTTTACTATATATAGCTA | 0.070 | 0.955 | - | - |
| 12409680 | - | 100 | 65.00 | 94.20 | 1 | 371382 | 371447 | TGCAGCAGGCAAATGCTTATACCTAGGCTGCTAC  TTAGGGACGTAGTCGTAGTCTAGGTTCTAGGTTGT | 0.091 | 0.960 | - | - |
| 12403800 | - | 100 | 68.00 | 98.55 | 7 | 1772634 | 1772702 | TGCAGGACGCGTGCCAAGCCAGAGTGGGGCCTGC  TTGACTTCGACTTTTGAGGAGCTCTGGCCAAACGA | 0.070 | 0.959 | - | - |
| 12389490 | - | 100 | 47.00 | 68.11 | 7 | 1772634 | 1772681 | TGCAGGACGCGTGCCAAGCCAGAGTGGGGCCTGC  TTGACTTCGACTTTGAGGAGCTCTGGCCAAACGAT | 0.927 | 0.040 | - | - |
| **12410413** | - | 100 | 53.00 | 76.81 | 7 | 1779092 | 1779145 | TGCAGCGTTCTCAACAGGGTCCTGATGGCAGGAGG  AACGAGAGGAGGGTTATTACAGATCGGAAGAGCG | 0.929 | 0.043 | - | - |
| 12410616 | - | two hits | 41 | 59.42 | 9 & 17(?) | ? | ? | TGCAGTATCTTTCTAACTAGACAAGACCTAACATT  ACTTATACTGTTACAGATCGGAAGAGCGGTTCAG | 1.000 | 0.022 | - | - |
| 12410844 | 12410844 | many hits | ? | ? | ? | ? | ? | TGCAGCTGCTAGAAAAGATAATAGCAGCGATGAT  AATAACGAGTGTAAATCAGATTCTAACTCGGAAGG | 1.000 | 0.022 | 0.947 | 0.078 |
| 12389607 | 12389607 | many hits | ? | ? | ? | ? | ? | TGCAGTAGTAGTAATAGCGCAGTTAGAAGTTAGA  TATTGTATTAGAGGTAAGGTTTACAGATCGGAAGA | 1.000 | 0.021 | 0.919 | 0.080 |
| 12406367 | - | many hits | Low | ? | ? | ? | ? | TGCAGTAACAATAAGGTGTTATATAGCTATGTAAA  TTACAGATCGGAAGAGCGGTTCAGCAGGAATGCC | 1.000 | 0.021 | - | - |
| 12389384 | 12389384 | 100 | 65.00 | 94.20 | 7 | 1879315 | 1879380 | TGCAGAGGTAGAATTCTTCCATTGCCTTCTCGCAA  TGCAAGGATATGCGCAAATGTAACATACATACAT | 1.000 | 0.021 | 0.925 | 0.078 |
| 12397726 | **12397726** | 100 | 65.00 | 94.20 | 7 | 1879787 | 1879852 | TGCAGGAGTATTACTTCGCGAAGCACCGGGTAGC  TTCGATGAGGTTGCTAGGCCGTCTAGAACCCGCGT | 1.000 | 0.020 | 0.947 | 0.060 |
| 12412057 | 12412057 | 100 | 68.00 | 98.55 | 7(?) | 1561581 | 1561649 | TGCAGGTTTTCTTTTAGTTCCTAGGTGTTTTCTAA  ATATAGGTATCCTTTCTATTTGATAAGGTATTTC | 0.000 | 1.000 | 0.000 | 0.961 |
| 12411328 | 12411328 | many hits | Low | ? | ? | ? | ? | TGCAGTTTGAAATATTCTACAATGCCATAGTTCTC  ATCGGCCATGCAATTACAGATCGGAAGAGCGGTT | 1.000 | 0.021 | 1.000 | 0.021 |
| 12389492 | 12389492 | 100 | 68.00 | 98.55 | 7 | 1959690 | 1959758 | TGCAGGAGAATCTTCCAGGCCATCAGGCTAGGGT  CTTCGCCGTTGATTGGTCGCCTGATGGAGAGCGTG | 1.000 | 0.021 | 1.000 | 0.060 |
| 12408335 | 12408335 | 100 | 68.00 | 98.55 | 7 | 2021725 | 2021793 | TGCAGCTCCCTCGACCGTAGTTCTAGTTATCTGTA  CAGAGCGGAGAATCTCTCGAGTCAGCGGTCGCCA | 0.976 | 0.021 | 1.000 | 0.021 |
| 12404162 | 12404162 | 100 | 68.00 | 98.55 | 7 | 2021928 | 2021996 | TGCAGCAGTTTTCCTGAACCGGGCTTCCGATGTGC  AGGCTTTCATGGAGCTCGGAGCTAATGCTTCTGG | 1.000 | 0.000 | 1.000 | 0.020 |
| 12407731 | 12407731 | 100 | 67.00 | 97.10 | 7 | 2021932 | 2021999 | TGCAGTTTTCCTGAACCGGGCTTCCGATATGCAGG  CTTTCATGGAGCTCGGAGCTAATGCTTCTGGCCG | 0.000 | 1.000 | 0.000 | 0.979 |
| 12389489 | 12389489 | 100 | 68.00 | 98.55 | 7 | 2053869 | 2053937 | TGCAGGAATGTGGCAGTTTCGGCTTAGAAATGCA  AGGAGGATTGTGTTTTCAGTCCGGCCGATCTTGTC | 1.000 | 0.020 | 1.000 | 0.039 |
| 12406518 | 12406518 | 100 | 68.00 | 98.55 | 7 | 2060399 | 2060467 | TGCAGAGCTGTCATCTCCTACCGACGGATGTGTCT  GGGAAAGGCGGTCAATGCTGGTGCATATGGCCAT | 0.000 | 1.000 | 0.000 | 0.960 |
| 12406517 | 12406517 | 100 | 68.00 | 98.55 | 7 | 2060399 | 2060467 | TGCAGCGCTGTCATCTCCTACCAACGGATGTGTCT  GGGAAAGGCGGTCAATGCTGGTGCATATGGCCAT | 1.000 | 0.021 | 1.000 | 0.020 |
| 12411995 | 12411995 | 100 | 68.00 | 98.55 | 7 | 2086738 | 2086806 | TGCAGAGGAGCTCGCACTCAAGCGGCAAGCACAT  GAGCAGATACGCAAGTGCATGGGCAAGATCTCGAA | 1.000 | 0.021 | 1.000 | 0.021 |
| 12400645 | 12400645 | 100 | 68.00 | 98.55 | 7 | 2093411 | 2093479 | TGCAGCGCACGCATTTGGCATCCTGGCTTCGCATC  CAAGCGCAAGAGCCCAGCAGCAACTTGCACAGCT | 1.000 | 0.020 | 1.000 | 0.000 |
| 12400691 | 12400691 | 100 | 68.00 | 98.55 | 7 | 2093476 | 2093544 | TGCAGTCATGCGACGACCATGGTGATTGGATTTGA  GTAAGGTTAGGAGAAACTCGGTCCGTCCAACGTG | 0.976 | 0.020 | 1.000 | 0.038 |
| 12389586 | 12389586 | 100 | 68.00 | 98.55 | 7 | 2096337 | 2096405 | TGCAGTTTGAGGGGAAGGATTGGTTTGCTTGCCCA  GTCGATGAATTCAACACTGGGTACAGCATCTGGG | 1.000 | 0.020 | 1.000 | 0.020 |
| 12409026 | 12409026 | 100 | 68.00 | 98.55 | 7 | 2116191 | 2116259 | TGCAGACTCCGACCAAGGCTAGAGTTATCACATAG  TAAACTAAATGGATCTGCGTGAATGGCTGCAAGT | 0.000 | 1.000 | 0.000 | 0.956 |
| 12401391 | 12401391 | 100 | 44.00 | 63.76 | 7 | 2119772 | 2119816 | TGCAGCGCACTACCAACGCAGCCACAGGCATCCC  TTTGTCTCGGTTACAGATCGGAAGAGCGGTTCAGC | 1.000 | 0.000 | 1.000 | 0.020 |
| **12405280** | 12405280 | 100 | 68.00 | 98.55 | 7 | 2130447 | 2130515 | TGCAGCTCGGTGGCTTCGTGGACGCACTTGAGATG  TTATGCTGCGGTTGAGTGAGAAGATATCAGAAGC | 1.000 | 0.065 | 0.974 | 0.060 |
| 12397704 | 12397704 | 100 | 57.00 | 82.60 | 6 (?) | 4164497 | 4164554 | TGCAGCTAAGAAGCCTACCGCCCCGAAGAACAAT  ACACTAGCTGCACCTAAGGTAGCTACTAGCAATAA | 1.000 | 0.061 | 1.000 | 0.020 |
| 12408748 | 12408748 | 100 | 45.00 | 65.21 | 7 | 2135277 | 2135321 | TGCAGTCAAGCAAGTGCAGACTTGGTCCATGGAAA  GAGCGGGTCTTACAGATCGGAAGAGCGGTTCAGC | 1.000 | 0.063 | 1.000 | 0.022 |
| 12406369 | 12406369 | No hit | ? | ? | ? | ? | ? | TGCAGTAGTAATAGCATTCAATTCAATATCTACTA  TATTACAGATCGGAAGAGCGGTTCAGCAGGAATG | 1.000 | 0.061 | 1.000 | 0.058 |
| 12410930 | 12410930 | many hits | ? | ? | ? | ? | ? | TGCAGGAATACGCAGCTCTCCTAGACGTCGGTAG  CTATTCCTAAAATTGCACTTAGGACAGCTCTTATA | 1.000 | 0.064 | 1.000 | 0.039 |
| 12408983 | 12408983 | many hits | ? | ? | ? | ? | ? | TGCAGTAGTGCCTGTTGCGACGGGTATAGGCGTCT  TAGTCGTATTATTCTTACAGATCGGAAGAGCGGT | 1.000 | 0.061 | 1.000 | 0.021 |
| 12408494 | 12408494 | many hits | Low score | ? | ? | ? | ? | TGCAGACCGGCCCGCTAGCGGCGCTATTACAGATC  GGAAGAGCGGTTCAGCAGGAATGCCGAGACCGAT | 1.000 | 0.061 | 1.000 | 0.021 |
| 12403131 | 12403131 | many hits | Low score | ? | ? | ? | ? | TGCAGTACTTTATAACAACGAAACAGCTTACAGAT  CGGAAGAGCGGTTCAGCAGGAATGCCGAGACCGA | 1.000 | 0.061 | 0.974 | 0.058 |
| 12406338 | 12406338 | two hits | Low score | ? | 1(?) | ? | ? | TGCAGTAAACCCGCCTACTTTTACAGATCGGAAGA  GCGGTTCAGCAGGAATGCCGAGACCGATCTCGTA | 1.000 | 0.061 | 1.000 | 0.059 |
| 12389524 | 12389524 | many hits | Low score | ? | ? | ? | ? | TGCAGGTAGTAGTAGTGGTTTTGGTTTCCGTCCTT  TAGTAGGTAGATAGGGTAGTAGTAAATTAGTATA | 0.974 | 0.060 | 1.000 | 0.020 |
| 12410210 | - | 100 | 66.00 | 95.65 | 5 (?) | 513760 | 513826 | TGCAGCCGGGAACGCGTACGGAGGGGCACCGTAC  TTGTTTGTAAGACTAGAGGGCGAGGTAGGGCTAGG | 1.000 | 0.086 | - | - |
| - | 12412318 | many hits | ? | ? | ? | ? | ? | TGCAGAACTGTTAGTAGATAATCAAAAAATTTCAA  AACCGAATAAAAAAGAAATAACTAGTATAGGAAT | - | - | 0.000 | 0.955 |
| 12388740 | 12388740 | 100 | 68.00 | 98.55 | 7 | 2145469 | 2145537 | TGCAGAACACTCCTGGGAACTCACATCTCAGCAT  CGTTGTTTTGCTGCGTAGAGAAACATGGGATGCGC | 0.000 | 0.956 | 0.000 | 0.929 |
| 12403524 | 12403524 | 100 | 68.00 | 98.55 | 7 | 2160240 | 2160308 | TGCAGAGTCAATCATAGTCGCGAGGAAAGCACAC  GAGAGGTAGATGTAGGTAATGATGCTCTGCTCTGC | 1.000 | 0.060 | 0.967 | 0.057 |
| 12396542 | 12396542 | 100 | 68.00 | 98.55 | 7 | 2163974 | 2164042 | TGCAGAAACTCAGAGTCATGCTTTTGGGCCGTCG  CCCTGGTTTTCATCAAGAGAGAACATTATACAATC | 0.000 | 0.951 | 0.000 | 0.957 |
| 12396543 | 12396543 | 100 | 68.00 | 98.55 | 7 | 2163974 | 2164042 | TGCAGAAGCTCAGAGTCATGCTTTTGGGCCGTCG  CCCTGGTTTTCATCAAGAGAGAACATTATATAATC | 1.000 | 0.060 | 1.000 | 0.038 |
| 12405725 | 12405725 | 100 | 68.00 | 98.55 | 7 | 2164326 | 2164394 | TGCAGAATCGAGACTTCTTTGCTTGACTCTACTTC  CTCAGGCGAGACAGCCGTGTTCGTGGCAAGCAAA | 0.000 | 0.959 | 0.000 | 0.957 |
| 12405724 | 12405724 | 100 | 68.00 | 98.55 | 7 | 2164326 | 2164394 | TGCAGAATCGAGACTTCTTTGCTTGACTCGACTTC  CTCAGGCGAGACGGCCGTGTTCGTGGCAAGCAAA | 1.000 | 0.065 | 1.000 | 0.020 |
| 12399874 | - | 100 | 68.00 | 98.55 | 7 | 2175183 | 2175251 | TGCAGCCCAACGCTCCGCCACCAGAGCAGGTTCGA  GCTCGGCAGGAGCAGGATCGGCAGCGTGAGCAGG | 0.068 | 0.957 | - | - |
| 12399875 | **12399875** | 100 | 68.00 | 98.55 | 7 | 2175183 | 2175251 | TGCAGCCCAACGTTCCGCCACCAGATCAGGTTCGA  GCTCGGCAGGAGCAGGATCGGCAGCGTGAGCAGG | 0.925 | 0.060 | 0.947 | 0.096 |
| 12406320 | - | many hits | Low score | ? | ? | ? | ? | TGCAGCAATTTCATCCTACTTACTTAGAGGTCCTT  TAGATATATTACAGATCGGAAGAGCGGTTCAGCA | 0.070 | 0.956 | - | - |
| 12389553 | 12389553 | many hits | Low score | ? | ? | ? | ? | TGCAGTCAGCGCAGATGGCCATCGATAAATTGATG  AGCGATAGGGCGGCCGAGAAAGCAGCTTATGAGA | 0.930 | 0.063 | 0.950 | 0.078 |
| - | 12406151 | 97 | 29.00 | 42.02 | 7 | 2177024 | 2177056 | TGCAGCTCTGTCGGTCCTTGCGCTGGGCTCCGAGA  CCGACGCTCGTCTCTCCGGAAGGAGCCATGGGTC | - | - | 0.950 | 0.082 |
| - | 12399094 | 93.9 | 25.00 | 36.23 | 7 | 2177225 | 2177257 | TGCAGCCTGGCGTCGAATCTCCTCTTCGGCCTTAC  AGATCGGAAGAGCGGTTCAGCAGGAATGCCGAGA | - | - | 0.950 | 0.080 |
| 12397650 | 12397650 | many hits | Low score | ? | ? | ? | ? | TGCAGAGCTCGCAGAAGAGCGGCGGGTGTACCAT  CTTGCTCTGCGCGAGATGGCGATGAGGAATCTTGC | 0.930 | 0.064 | 0.950 | 0.082 |
| 12403967 | - | 100 | 68.00 | 98.55 | 7 | 2207215 | 2207283 | TGCAGTCCTGTATGGAGCCAGCAGATGGCTTATTA  CACCAATGGTAGACGCGCTATCTGAGTCGCGACA | 0.140 | 0.909 | - | - |
