# Supplementary material for: Pfcyp51 exclusively determines reduced sensitivity to 14α-demethylase inhibitor fungicides in the banana black Sigatoka pathogen Pseudocercospora fijiensis
Source: PLoS One. 2019 Oct 17;14(10):e0223858. doi: 10.1371/journal.pone.0223858 (PMC6797121; doi:10.1371/journal.pone.0223858)
Supplement: S2 Table — *Strains with scores <0.2 mg.l-1 were classified as sensitive and strains with scores >1.00 mg.l-1 were classified as resistant. Strains with question marks (?) remained undetermined. (DOCX) [file pone.0223858.s007.docx]

| **Number** | **Strain** | **Insertions in the promoter of the *cyp51* gene** | **Amino acid CYP51 substitutions** | | | | | | | | **DMI fungicide EC_50_ average scores*** | | |
| --- | --- | --- | --- | --- | --- | --- | --- | --- | --- | --- | --- | --- | --- |
|  |  | **Promoter** | **T18** | **V106** | **Y136** | **A313** | **H380** | **A381** | **Y463** | **A446** | **Difenoconazole** | **Epoxiconazole** | **Propiconazole** |
| Ref_1 | Bo_1 | WT | T18I | V106D | WT | WT | WT | WT | WT | A446S | 0.006 | 0.013 | 0.018 |
| Ref_2 | CaM10_6 | AAATCTCGT ACGATAGCAT AAAATCTCGT ACGATAGCAT AAAATCTCGT ACGATGTTAA ATCTCGTACG ATAGCATAAA TCTCGTACGA TAGCACCTGC CCAT | T18I | V106D | Y136F | A313G | WT | WT | Y463D | WT | 17.194 | 7.496 | 11.750 |
| 1 | N2_1 | AAATCTCGT ACGATAGCAT AAAATCTCGT ACGATAGCAT AAAATCTCGT ACGATGTTAA ATCTCGTACG ATAGCATAAA TCTCGTACGA TAGCACCTGC CCAT | T18I | V106D | Y136F | A313G | WT | WT | Y463D | WT | >10.24 | 5.036 | >10.24 |
| 2 | N2_2 | AAATCTCGT ACGATAGCAT AAAATCTCGT ACGATAGCAT AAAATCTCGT ACGATGTTAA ATCTCGTACG ATAGCATAAA TCTCGTACGA TAGCACCTGC CCAT | T18I | V106D | Y136F | A313G | WT | WT | Y463D | WT | 9.086 | 6.886 | 5.521 |
| 3 | N2_3 | AAATCTCGT ACGATAGCAT AAAATCTCGT ACGATAGCAT AAAATCTCGT ACGATGTTAA ATCTCGTACG ATAGCATAAA TCTCGTACGA TAGCACCTGC CCAT | T18I | V106D | Y136F | A313G | WT | WT | Y463D | WT | 7.900 | 4.059 | 6.082 |
| 4 | N2_4 | WT | T18I | V106D | WT | WT | WT | WT | WT | A446S | 0.017 | 0.026 | 0.052 |
| 5 | N2_5 | AAATCTCGT ACGATAGCAT AAAATCTCGT ACGATAGCAT AAAATCTCGT ACGATGTTAA ATCTCGTACG ATAGCATAAA TCTCGTACGA TAGCACCTGC CCAT | T18I | V106D | Y136F | A313G | WT | WT | Y463D | WT | 9.420 | 3.693 | 6.392 |
| 6 | N2_6 | AAATCTCGT ACGATAGCAT AAAATCTCGT ACGATAGCAT AAAATCTCGT ACGATGTTAA ATCTCGTACG ATAGCATAAA TCTCGTACGA TAGCACCTGC CCAT | T18I | V106D | Y136F | A313G | WT | WT | Y463D | WT | 8.313 | 5.966 | 5.958 |
| 7 | N2_7 | WT | T18I | V106D | WT | WT | WT | WT | WT | A446S | 0.099 | 0.089 | 0.102 |
| 8 | N2_8 | WT | T18I | V106D | WT | WT | WT | WT | WT | A446S | 0.014 | 0.016 | 0.034 |
| 9 | N2_9 | WT | T18I | V106D | WT | WT | WT | WT | WT | A446S | 0.028 | 0.036 | 0.069 |
| 10 | N2_10 | AAATCTCGT ACGATAGCAT AAAATCTCGT ACGATAGCAT AAAATCTCGT ACGATGTTAA ATCTCGTACG ATAGCATAAA TCTCGTACGA TAGCACCTGC CCAT | T18I | V106D | Y136F | A313G | WT | WT | Y463D | WT | 8.749 | 4.855 | >10.24 |
| 11 | N2_11 | AAATCTCGT ACGATAGCAT AAAATCTCGT ACGATAGCAT AAAATCTCGT ACGATGTTAA ATCTCGTACG ATAGCATAAA TCTCGTACGA TAGCACCTGC CCAT | T18I | V106D | Y136F | A313G | WT | WT | Y463D | WT | >10.24 | 5.553 | >10.24 |
| 12 | N2_12 | AAATCTCGT ACGATAGCAT AAAATCTCGT ACGATAGCAT AAAATCTCGT ACGATGTTAA ATCTCGTACG ATAGCATAAA TCTCGTACGA TAGCACCTGC CCAT | T18I | V106D | Y136F | A313G | WT | WT | Y463D | WT | 6.528 | 2.690 | 5.385 |
| 13 | N2_13 | ? | ? | ? | ? | ? | ? | ? | ? | ? | 7.994 | 2.320 | 6.199 |
| 14 | N2_14 | WT | T18I | V106D | WT |  | WT | WT | WT | A446S | 0.037 | 0.064 | 0.070 |
| 15 | N2_15 | WT | T18I | V106D | WT | WT | WT | WT | WT | A446S | 0.034 | 0.052 | 0.056 |
| 16 | N2_16 | WT | T18I | V106D | WT | WT | WT | WT | WT | A446S | 0.017 | 0.025 | 0.057 |
| 17 | N2_17 | WT | T18I | V106D | WT | WT | WT | WT | WT | A446S | 0.059 | 0.069 | 0.073 |
| 18 | N2_18 | AAATCTCGT ACGATAGCAT AAAATCTCGT ACGATAGCAT AAAATCTCGT ACGATGTTAA ATCTCGTACG ATAGCATAAA TCTCGTACGA TAGCACCTGC CCAT | T18I | V106D | Y136F | A313G | WT | WT | Y463D | WT | 8.474 | 1.995 | 8.059 |
| 19 | N2_19 | AAATCTCGT ACGATAGCAT AAAATCTCGT ACGATAGCAT AAAATCTCGT ACGATGTTAA ATCTCGTACG ATAGCATAAA TCTCGTACGA TAGCACCTGC CCAT | T18I | V106D | Y136F | A313G | WT | WT | Y463D | WT | >10.24 | 4.332 | >10.24 |
| 20 | N2_20 | AAATCTCGT ACGATAGCAT AAAATCTCGT ACGATAGCAT AAAATCTCGT ACGATGTTAA ATCTCGTACG ATAGCATAAA TCTCGTACGA TAGCACCTGC CCAT | T18I | V106D | Y136F | A313G | WT | WT | Y463D | WT | 7.132 | 1.894 | 8.337 |
| 21 | N2_21 | WT | T18I | V106D | WT | WT | WT | WT | WT | A446S | 0.213 | 0.111 | 0.120 |
| 22 | N2_22 | WT | T18I | V106D | WT | WT | WT | WT | WT | A446S | 0.022 | 0.021 | 0.063 |
| 23 | N2_23 | AAATCTCGT ACGATAGCAT AAAATCTCGT ACGATAGCAT AAAATCTCGT ACGATGTTAA ATCTCGTACG ATAGCATAAA TCTCGTACGA TAGCACCTGC CCAT | T18I | V106D | Y136F | A313G | WT | WT | Y463D | WT | 8.459 | 3.239 | 6.902 |
| 24 | N2_24 | WT | T18I | V106D | WT | WT | WT | WT | WT | A446S | 0.010 | 0.015 | 0.027 |
| 25 | N2_25 | WT | T18I | V106D | WT |  | WT | WT | WT | A446S | 0.094 | 0.086 | 0.081 |
| 26 | N2_26 | WT | T18I | V106D | WT | WT | WT | WT | WT | A446S | 0.091 | 0.036 | 0.090 |
| 27 | N2_27 | AAATCTCGT ACGATAGCAT AAAATCTCGT ACGATAGCAT AAAATCTCGT ACGATGTTAA ATCTCGTACG ATAGCATAAA TCTCGTACGA TAGCACCTGC CCAT | T18I | V106D | Y136F | A313G | WT | WT | Y463D | WT | 9.589 | 7.057 | 8.697 |
| 28 | N2_28 | WT | T18I | V106D | WT | WT | WT | WT | WT | A446S | 0.061 | 0.036 | 0.074 |
| 29 | N2_29 | AAATCTCGT ACGATAGCAT AAAATCTCGT ACGATAGCAT AAAATCTCGT ACGATGTTAA ATCTCGTACG ATAGCATAAA TCTCGTACGA TAGCACCTGC CCAT | T18I | V106D | Y136F | A313G | WT | WT | Y463D | WT | 8.274 | 4.781 | 6.005 |
| 30 | N2_30 | AAATCTCGT ACGATAGCAT AAAATCTCGT ACGATAGCAT AAAATCTCGT ACGATGTTAA ATCTCGTACG ATAGCATAAA TCTCGTACGA TAGCACCTGC CCAT | T18I | V106D | Y136F | A313G | WT | WT | Y463D | WT | 8.064 | 3.219 | 7.893 |
| 31 | N2_31 | WT | T18I | V106D | WT | WT | WT | WT | WT | A446S | 0.032 | 0.024 | 0.080 |
| 32 | N2_32 | WT | T18I | V106D | WT | WT | WT | WT | WT | A446S | 0.042 | 0.031 | 0.077 |
| 33 | N2_33 | WT | T18I | V106D | WT | WT | WT | WT | WT | A446S | 0.082 | 0.073 | 0.088 |
| 34 | N2_34 | WT | T18I | V106D | WT | WT | WT | WT | WT | A446S | 0.017 | 0.018 | 0.065 |
| 35 | N2_35 | WT | T18I | V106D | WT | WT | WT | WT | WT | A446S | 0.099 | 0.190 | 0.135 |
| 36 | N2_36 | WT | T18I | V106D | WT | WT | WT | WT | WT | A446S | 0.019 | 0.020 | 0.055 |
| 37 | N2_37 | AAATCTCGT ACGATAGCAT AAAATCTCGT ACGATAGCAT AAAATCTCGT ACGATGTTAA ATCTCGTACG ATAGCATAAA TCTCGTACGA TAGCACCTGC CCAT | T18I | V106D | Y136F | A313G | WT | WT | Y463D | WT | 6.654 | 2.838 | 6.327 |
| 38 | N2_38 | AAATCTCGT ACGATAGCAT AAAATCTCGT ACGATAGCAT AAAATCTCGT ACGATGTTAA ATCTCGTACG ATAGCATAAA TCTCGTACGA TAGCACCTGC CCAT | T18I | V106D | Y136F | A313G | WT | WT | Y463D | WT | >10.24 | >10.24 | >10.24 |
| 39 | N2_39 | AAATCTCGT ACGATAGCAT AAAATCTCGT ACGATAGCAT AAAATCTCGT ACGATGTTAA ATCTCGTACG ATAGCATAAA TCTCGTACGA TAGCACCTGC CCAT | T18I | V106D | Y136F | A313G | WT | WT | Y463D | WT | 9.127 | 4.996 | 4.463 |
| 40 | N2_40 | AAATCTCGT ACGATAGCAT AAAATCTCGT ACGATAGCAT AAAATCTCGT ACGATGTTAA ATCTCGTACG ATAGCATAAA TCTCGTACGA TAGCACCTGC CCAT | T18I | V106D | Y136F | A313G | WT | WT | Y463D | WT | 9.310 | 3.568 | 9.174 |
| 41 | N2_41 | WT | T18I | V106D | WT | WT | WT | WT | WT | A446S | 0.009 | 0.018 | 0.048 |
| 42 | N2_42 | WT | T18I | V106D | WT | WT | WT | WT | WT | A446S | 0.013 | 0.018 | 0.036 |
| 43 | N2_43 | WT | T18I | V106D | WT | WT | WT | WT | WT | A446S | 0.013 | 0.018 | 0.028 |
| 44 | N2_44 | WT | T18I | V106D | WT | WT | WT | WT | WT | A446S | 0.068 | 0.061 | 0.073 |
| 45 | N2_45 | AAATCTCGT ACGATAGCAT AAAATCTCGT ACGATAGCAT AAAATCTCGT ACGATGTTAA ATCTCGTACG ATAGCATAAA TCTCGTACGA TAGCACCTGC CCAT | T18I | V106D | Y136F | A313G | WT | WT | Y463D | WT | 10.072 | 5.651 | >10.24 |
| 46 | N2_46 | AAATCTCGT ACGATAGCAT AAAATCTCGT ACGATAGCAT AAAATCTCGT ACGATGTTAA ATCTCGTACG ATAGCATAAA TCTCGTACGA TAGCACCTGC CCAT | T18I | V106D | Y136F | A313G | WT | WT | Y463D | WT | >10.24 | 5.014 | >10.24 |
| 47 | N2_47 | WT | T18I | V106D | WT | WT | WT | WT | WT | A446S | 0.031 | 0.021 | 0.057 |
| 48 | N2_48 | AAATCTCGT ACGATAGCAT AAAATCTCGT ACGATAGCAT AAAATCTCGT ACGATGTTAA ATCTCGTACG ATAGCATAAA TCTCGTACGA TAGCACCTGC CCAT | T18I | V106D | Y136F | A313G | WT | WT | Y463D | WT | 8.675 | 2.173 | 9.186 |
| 49 | N2_49 | AAATCTCGT ACGATAGCAT AAAATCTCGT ACGATAGCAT AAAATCTCGT ACGATGTTAA ATCTCGTACG ATAGCATAAA TCTCGTACGA TAGCACCTGC CCAT | T18I | V106D | Y136F | A313G | WT | WT | Y463D | WT | 8.196 | 5.808 | >10.24 |
| 50 | N2_50 | AAATCTCGT ACGATAGCAT AAAATCTCGT ACGATAGCAT AAAATCTCGT ACGATGTTAA ATCTCGTACG ATAGCATAAA TCTCGTACGA TAGCACCTGC CCAT | T18I | V106D | Y136F | A313G | WT | WT | Y463D | WT | 10.142 | 2.202 | 10.051 |
| 51 | N2_51 | WT | T18I | V106D | WT | WT | WT | WT | WT | A446S | 0.030 | 0.018 | 0.059 |
| 52 | N2_52 | AAATCTCGT ACGATAGCAT AAAATCTCGT ACGATAGCAT AAAATCTCGT ACGATGTTAA ATCTCGTACG ATAGCATAAA TCTCGTACGA TAGCACCTGC CCAT | T18I | V106D | Y136F | A313G | WT | WT | Y463D | WT | >10.24 | 6.359 | >10.24 |
| 53 | N2_53 | AAATCTCGT ACGATAGCAT AAAATCTCGT ACGATAGCAT AAAATCTCGT ACGATGTTAA ATCTCGTACG ATAGCATAAA TCTCGTACGA TAGCACCTGC CCAT | T18I | V106D | Y136F | A313G | WT | WT | Y463D | WT | 7.049 | 5.746 | 6.272 |
| 54 | N2_54 | WT | T18I | V106D | WT | WT | WT | WT | WT | A446S | 0.068 | 0.056 | 0.082 |
| 55 | N2_55 | WT | T18I | V106D | WT | WT | WT | WT | WT | A446S | 0.045 | 0.058 | 0.076 |
| 56 | N2_56 | AAATCTCGT ACGATAGCAT AAAATCTCGT ACGATAGCAT AAAATCTCGT ACGATGTTAA ATCTCGTACG ATAGCATAAA TCTCGTACGA TAGCACCTGC CCAT | T18I | V106D | Y136F | A313G | WT | WT | Y463D | WT | >10.24 | 8.699 | 9.446 |
| 57 | N2_57 | WT | T18I | V106D | WT | WT | WT | WT | WT | A446S | 0.091 | 0.066 | 0.101 |
| 58 | N2_58 | WT | T18I | V106D | WT | WT | WT | WT | WT | A446S | 0.087 | 0.058 | 0.102 |
| 59 | N2_59 | WT | T18I | V106D | WT | WT | WT | WT | WT | A446S | 0.078 | 0.073 | 0.077 |
| 60 | N2_60 | AAATCTCGT ACGATAGCAT AAAATCTCGT ACGATAGCAT AAAATCTCGT ACGATGTTAA ATCTCGTACG ATAGCATAAA TCTCGTACGA TAGCACCTGC CCAT | T18I | V106D | Y136F | A313G | WT | WT | Y463D | WT | >10.24 | 3.645 | >10.24 |
| 61 | N2_61 | AAATCTCGT ACGATAGCAT AAAATCTCGT ACGATAGCAT AAAATCTCGT ACGATGTTAA ATCTCGTACG ATAGCATAAA TCTCGTACGA TAGCACCTGC CCAT | T18I | V106D | Y136F | A313G | WT | WT | Y463D | WT | 8.646 | 6.709 | 7.779 |
| 62 | N2_62 | AAATCTCGT ACGATAGCAT AAAATCTCGT ACGATAGCAT AAAATCTCGT ACGATGTTAA ATCTCGTACG ATAGCATAAA TCTCGTACGA TAGCACCTGC CCAT | T18I | V106D | Y136F | A313G | WT | WT | Y463D | WT | >10.24 | 3.105 | >10.24 |
| 63 | N2_63 | AAATCTCGT ACGATAGCAT AAAATCTCGT ACGATAGCAT AAAATCTCGT ACGATGTTAA ATCTCGTACG ATAGCATAAA TCTCGTACGA TAGCACCTGC CCAT | T18I | V106D | Y136F | A313G | WT | WT | Y463D | WT | 9.886 | 2.980 | 7.598 |
| 64 | N2_64 | WT | T18I | V106D | WT | WT | WT | WT | WT | A446S | 0.063 | 0.017 | 0.075 |
| 65 | N2_65? | ? | ? | ? | ? | ? | ? | ? | ? | ? | 7.457 | 1.351 | 3.887 |
| 66 | N2_66 | AAATCTCGT ACGATAGCAT AAAATCTCGT ACGATAGCAT AAAATCTCGT ACGATGTTAA ATCTCGTACG ATAGCATAAA TCTCGTACGA TAGCACCTGC CCAT | T18I | V106D | Y136F | A313G | WT | WT | Y463D | WT | >10.24 | 7.392 | 8.710 |
| 67 | N2_67 | WT | T18I | V106D | WT | WT | WT | WT | WT | A446S | 0.014 | 0.015 | 0.027 |
| 68 | N2_68 | AAATCTCGT ACGATAGCAT AAAATCTCGT ACGATAGCAT AAAATCTCGT ACGATGTTAA ATCTCGTACG ATAGCATAAA TCTCGTACGA TAGCACCTGC CCAT | T18I | V106D | Y136F | A313G | WT | WT | Y463D | WT | 7.430 | 4.047 | 5.682 |
| 69 | N2_69 | WT | T18I | V106D | WT |  | WT | WT | WT | A446S | 0.025 | 0.018 | 0.067 |
| 70 | N2_70 | AAATCTCGT ACGATAGCAT AAAATCTCGT ACGATAGCAT AAAATCTCGT ACGATGTTAA ATCTCGTACG ATAGCATAAA TCTCGTACGA TAGCACCTGC CCAT | T18I | V106D | Y136F | A313G | WT | WT | Y463D | WT | 8.125 | 6.326 | 6.596 |
| 71 | N2_71 | AAATCTCGT ACGATAGCAT AAAATCTCGT ACGATAGCAT AAAATCTCGT ACGATGTTAA ATCTCGTACG ATAGCATAAA TCTCGTACGA TAGCACCTGC CCAT | T18I | V106D | Y136F | A313G | WT | WT | Y463D | WT | 7.181 | 2.295 | 6.488 |
| 72 | N2_72 | WT | T18I | V106D | WT | WT | WT | WT | WT | A446S | 0.025 | 0.020 | 0.068 |
| 73 | N2_73 | AAATCTCGT ACGATAGCAT AAAATCTCGT ACGATAGCAT AAAATCTCGT ACGATGTTAA ATCTCGTACG ATAGCATAAA TCTCGTACGA TAGCACCTGC CCAT | T18I | V106D | Y136F | A313G | WT | WT | Y463D | WT | 8.169 | 7.973 | 8.452 |
| 74 | N2_74 | AAATCTCGT ACGATAGCAT AAAATCTCGT ACGATAGCAT AAAATCTCGT ACGATGTTAA ATCTCGTACG ATAGCATAAA TCTCGTACGA TAGCACCTGC CCAT | T18I | V106D | Y136F | A313G | WT | WT | Y463D | WT | >10.24 | 7.302 | 8.374 |
| 75 | N2_75 | AAATCTCGT ACGATAGCAT AAAATCTCGT ACGATAGCAT AAAATCTCGT ACGATGTTAA ATCTCGTACG ATAGCATAAA TCTCGTACGA TAGCACCTGC CCAT | T18I | V106D | Y136F | A313G | WT | WT | Y463D | WT | 7.564 | 5.258 | 4.859 |
| 76 | N2_76 | AAATCTCGT ACGATAGCAT AAAATCTCGT ACGATAGCAT AAAATCTCGT ACGATGTTAA ATCTCGTACG ATAGCATAAA TCTCGTACGA TAGCACCTGC CCAT | T18I | V106D | Y136F | A313G | WT | WT | Y463D | WT | 5.669 | 1.612 | 4.982 |
| 77 | N2_77 | WT | T18I | V106D | WT | WT | WT | WT | WT | A446S | 0.025 | 0.023 | 0.080 |
| 78 | N2_78 | AAATCTCGT ACGATAGCAT AAAATCTCGT ACGATAGCAT AAAATCTCGT ACGATGTTAA ATCTCGTACG ATAGCATAAA TCTCGTACGA TAGCACCTGC CCAT | T18I | V106D | Y136F | A313G | WT | WT | Y463D | WT | 9.560 | 6.697 | 7.277 |
| 79 | N2_79 | WT | T18I | V106D | WT | WT | WT | WT | WT | A446S | 0.093 | 0.078 | 0.095 |
| 80 | N2_80 | WT | T18I | V106D | WT |  | WT | WT | WT | A446S | 0.032 | 0.020 | 0.080 |
| 81 | N2_81 | AAATCTCGT ACGATAGCAT AAAATCTCGT ACGATAGCAT AAAATCTCGT ACGATGTTAA ATCTCGTACG ATAGCATAAA TCTCGTACGA TAGCACCTGC CCAT | T18I | V106D | Y136F | A313G | WT | WT | Y463D | WT | >10.24 | 2.780 | >10.24 |
| 82 | N2_82 | AAATCTCGT ACGATAGCAT AAAATCTCGT ACGATAGCAT AAAATCTCGT ACGATGTTAA ATCTCGTACG ATAGCATAAA TCTCGTACGA TAGCACCTGC CCAT | T18I | V106D | Y136F | A313G | WT | WT | Y463D | WT | 7.223 | 4.711 | 6.111 |
| 83 | N2_83 | WT | T18I | V106D | WT | WT | WT | WT | WT | A446S | 0.015 | 0.017 | 0.031 |
| 84 | N2_84 | WT | T18I | V106D | WT |  | WT | WT | WT | A446S | 0.023 | 0.027 | 0.072 |
| 85 | N2_85 | AAATCTCGT ACGATAGCAT AAAATCTCGT ACGATAGCAT AAAATCTCGT ACGATGTTAA ATCTCGTACG ATAGCATAAA TCTCGTACGA TAGCACCTGC CCAT | T18I | V106D | Y136F | A313G | WT | WT | Y463D | WT | 7.696 | 2.712 | 8.177 |
| 86 | N2_86 | WT | T18I | V106D | WT | WT | WT | WT | WT | A446S | 0.025 | 0.020 | 0.068 |
| 87 | N2_87 | AAATCTCGT ACGATAGCAT AAAATCTCGT ACGATAGCAT AAAATCTCGT ACGATGTTAA ATCTCGTACG ATAGCATAAA TCTCGTACGA TAGCACCTGC CCAT | T18I | V106D | Y136F | A313G | WT | WT | Y463D | WT | 7.786 | 3.531 | 6.172 |
| 88 | N2_88 | WT | T18I | V106D | WT | WT | WT | WT | WT | A446S | 0.026 | 0.018 | 0.080 |
| 89 | N2_89 | WT | T18I | V106D | WT | WT | WT | WT | WT | A446S | 0.112 | 0.154 | 0.210 |
| 90 | N2_90 | AAATCTCGT ACGATAGCAT AAAATCTCGT ACGATAGCAT AAAATCTCGT ACGATGTTAA ATCTCGTACG ATAGCATAAA TCTCGTACGA TAGCACCTGC CCAT | T18I | V106D | Y136F | A313G | WT | WT | Y463D | WT | 7.799 | 5.684 | 3.958 |
| 91 | N2_91 | AAATCTCGT ACGATAGCAT AAAATCTCGT ACGATAGCAT AAAATCTCGT ACGATGTTAA ATCTCGTACG ATAGCATAAA TCTCGTACGA TAGCACCTGC CCAT | T18I | V106D | Y136F | A313G | WT | WT | Y463D | WT | >10.24 | 5.969 | >10.24 |
| 92 | N2_92 | WT | T18I | V106D | WT | WT | WT | WT | WT | A446S | 0.022 | 0.021 | 0.067 |
| 93 | N2_93 | WT | T18I | V106D | WT | WT | WT | WT | WT | A446S | 0.011 | 0.019 | 0.046 |
| 94 | N2_94 | AAATCTCGT ACGATAGCAT AAAATCTCGT ACGATAGCAT AAAATCTCGT ACGATGTTAA ATCTCGTACG ATAGCATAAA TCTCGTACGA TAGCACCTGC CCAT | T18I | V106D | Y136F | A313G | WT | WT | Y463D | WT | >10.24 | 5.477 | >10.24 |
| 95 | N2_95 | AAATCTCGT ACGATAGCAT AAAATCTCGT ACGATAGCAT AAAATCTCGT ACGATGTTAA ATCTCGTACG ATAGCATAAA TCTCGTACGA TAGCACCTGC CCAT | T18I | V106D | Y136F | A313G | WT | WT | Y463D | WT | >10.24 | 9.276 | >10.24 |
| 96 | N2_96 | AAATCTCGT ACGATAGCAT AAAATCTCGT ACGATAGCAT AAAATCTCGT ACGATGTTAA ATCTCGTACG ATAGCATAAA TCTCGTACGA TAGCACCTGC CCAT | T18I | V106D | Y136F | A313G | WT | WT | Y463D | WT | >10.24 | 7.419 | >10.24 |
| 97 | N2_97 | AAATCTCGT ACGATAGCAT AAAATCTCGT ACGATAGCAT AAAATCTCGT ACGATGTTAA ATCTCGTACG ATAGCATAAA TCTCGTACGA TAGCACCTGC CCAT | T18I | V106D | Y136F | A313G | WT | WT | Y463D | WT | 7.248 | 2.563 | 3.732 |
| 98 | N2_98 | WT | T18I | V106D | WT | WT | WT | WT | WT | A446S | 0.015 | 0.022 | 0.068 |
| 99 | N2_99 | AAATCTCGT ACGATAGCAT AAAATCTCGT ACGATAGCAT AAAATCTCGT ACGATGTTAA ATCTCGTACG ATAGCATAAA TCTCGTACGA TAGCACCTGC CCAT | T18I | V106D | Y136F | A313G | WT | WT | Y463D | WT | 8.764 | 5.704 | >>10.24 |
| 100 | N2_100 | WT | T18I | V106D | WT | WT | WT | WT | WT | A446S | 0.072 | 0.067 | 0.113 |
| Ref_3 | CaM10_21 | AAATCTCGT ACGATAGCAT AAAATCTCGT ACGATAGCAT AAAATCTCGT ACGATGTTAA ATCTCGTACG ATAGCATAAA TCTCGTACGA TAGCACCTGC CCAT | T18I | V106D | WT | WT | H380N | A381G | Y463D | WT | 7.871 | 10.054 | 6.349 |
| 101 | N5_1 | AAATCTCGT ACGATAGCAT AAAATCTCGT ACGATAGCAT AAAATCTCGT ACGATGTTAA ATCTCGTACG ATAGCATAAA TCTCGTACGA TAGCACCTGC CCAT | T18I | V106D | WT | WT | H380N | A381G | Y463D | WT | 0.597 | 0.748 | 0.683 |
| 102 | N5_2 | WT | T18I | V106D | WT | WT | WT | WT | WT | A446S | 0.079 | 0.142 | 0.157 |
| 103 | N5_3 | AAATCTCGT ACGATAGCAT AAAATCTCGT ACGATAGCAT AAAATCTCGT ACGATGTTAA ATCTCGTACG ATAGCATAAA TCTCGTACGA TAGCACCTGC CCAT | T18I | V106D | WT | WT | H380N | A381G | Y463D | WT | 2.104 | 3.162 | 4.616 |
| 104 | N5_4 | WT | T18I | V106D | WT |  | WT | WT | WT | A446S | 0.020 | 0.035 | 0.069 |
| 105 | N5_5 | AAATCTCGT ACGATAGCAT AAAATCTCGT ACGATAGCAT AAAATCTCGT ACGATGTTAA ATCTCGTACG ATAGCATAAA TCTCGTACGA TAGCACCTGC CCAT | T18I | V106D | WT | WT | H380N | A381G | Y463D | WT | 2.589 | 5.128 | 2.931 |
| 106 | N5_6 | AAATCTCGT ACGATAGCAT AAAATCTCGT ACGATAGCAT AAAATCTCGT ACGATGTTAA ATCTCGTACG ATAGCATAAA TCTCGTACGA TAGCACCTGC CCAT | T18I | V106D | WT | WT | H380N | A381G | Y463D | WT | 5.675 | 6.923 | 6.329 |
| 107 | N5_7 | WT | T18I | V106D | WT | WT | WT | WT | WT | A446S | 0.067 | 0.073 | 0.084 |
| 108 | N5_8 | WT | T18I | V106D | WT | WT | WT | WT | WT | A446S | 0.011 | 0.015 | 0.031 |
| 109 | N5_9 | AAATCTCGT ACGATAGCAT AAAATCTCGT ACGATAGCAT AAAATCTCGT ACGATGTTAA ATCTCGTACG ATAGCATAAA TCTCGTACGA TAGCACCTGC CCAT | T18I | V106D | WT | WT | H380N | A381G | Y463D | WT | 5.140 | 6.041 | 5.197 |
| 110 | N5_10 | WT | T18I | V106D | WT | WT | WT | WT | WT | A446S | 0.011 | 0.015 | 0.032 |
| 111 | N5_11 | AAATCTCGT ACGATAGCAT AAAATCTCGT ACGATAGCAT AAAATCTCGT ACGATGTTAA ATCTCGTACG ATAGCATAAA TCTCGTACGA TAGCACCTGC CCAT | T18I | V106D | WT | WT | H380N | A381G | Y463D | WT | 7.527 | 7.976 | 7.058 |
| 112 | N5_12 | AAATCTCGT ACGATAGCAT AAAATCTCGT ACGATAGCAT AAAATCTCGT ACGATGTTAA ATCTCGTACG ATAGCATAAA TCTCGTACGA TAGCACCTGC CCAT | T18I | V106D | WT | WT | H380N | A381G | Y463D | WT | 6.973 | 8.439 | 7.132 |
| 113 | N5_13 | WT | T18I | V106D | WT | WT | WT | WT | WT | A446S | 0.047 | 0.060 | 0.066 |
| 114 | N5_14 | AAATCTCGT ACGATAGCAT AAAATCTCGT ACGATAGCAT AAAATCTCGT ACGATGTTAA ATCTCGTACG ATAGCATAAA TCTCGTACGA TAGCACCTGC CCAT | T18I | V106D | WT | WT | H380N | A381G | Y463D | WT | 1.556 | 1.717 | 1.323 |
| 115 | N5_15 | WT | T18I | V106D | WT | WT | WT | WT | WT | A446S | 0.047 | 0.081 | 0.077 |
| 116 | N5_16 | AAATCTCGT ACGATAGCAT AAAATCTCGT ACGATAGCAT AAAATCTCGT ACGATGTTAA ATCTCGTACG ATAGCATAAA TCTCGTACGA TAGCACCTGC CCAT | T18I | V106D | WT | WT | H380N | A381G | Y463D | WT | 6.259 | 8.082 | 3.523 |
| 117 | N5_17 | AAATCTCGT ACGATAGCAT AAAATCTCGT ACGATAGCAT AAAATCTCGT ACGATGTTAA ATCTCGTACG ATAGCATAAA TCTCGTACGA TAGCACCTGC CCAT | T18I | V106D | WT | WT | H380N | A381G | Y463D | WT | 6.120 | 6.266 | 3.562 |
| 118 | N5_18 | WT | T18I | V106D | WT | WT | WT | WT | WT | A446S | 0.010 | 0.011 | 0.032 |
| 119 | N5_19 | AAATCTCGT ACGATAGCAT AAAATCTCGT ACGATAGCAT AAAATCTCGT ACGATGTTAA ATCTCGTACG ATAGCATAAA TCTCGTACGA TAGCACCTGC CCAT | T18I | V106D | WT | WT | H380N | A381G | Y463D | WT | 3.355 | 6.146 | 2.787 |
| 120 | N5_20 | WT | T18I | V106D | WT | WT | WT | WT | WT | A446S | 0.012 | 0.016 | 0.052 |
| 121 | N5_21 | AAATCTCGT ACGATAGCAT AAAATCTCGT ACGATAGCAT AAAATCTCGT ACGATGTTAA ATCTCGTACG ATAGCATAAA TCTCGTACGA TAGCACCTGC CCAT | T18I | V106D | WT | WT | H380N | A381G | Y463D | WT | 1.278 | 2.714 | 1.135 |
| 122 | N5_22 | AAATCTCGT ACGATAGCAT AAAATCTCGT ACGATAGCAT AAAATCTCGT ACGATGTTAA ATCTCGTACG ATAGCATAAA TCTCGTACGA TAGCACCTGC CCAT | T18I | V106D | WT | WT | H380N | A381G | Y463D | WT | 1.683 | 1.875 | 1.793 |
| 123 | N5_23 | AAATCTCGT ACGATAGCAT AAAATCTCGT ACGATAGCAT AAAATCTCGT ACGATGTTAA ATCTCGTACG ATAGCATAAA TCTCGTACGA TAGCACCTGC CCAT | T18I | V106D | WT | WT | H380N | A381G | Y463D | WT | 1.658 | 3.687 | 2.518 |
| 124 | N5_24 | WT | T18I | V106D | WT | WT | WT | WT | WT | A446S | 0.086 | 0.090 | 0.072 |
| 125 | N5_25 | AAATCTCGT ACGATAGCAT AAAATCTCGT ACGATAGCAT AAAATCTCGT ACGATGTTAA ATCTCGTACG ATAGCATAAA TCTCGTACGA TAGCACCTGC CCAT | T18I | V106D | WT | WT | H380N | A381G | Y463D | WT | 3.139 | 5.592 | 3.953 |
| 126 | N5_26 | WT | T18I | V106D | WT | WT | WT | WT | WT | A446S | 0.010 | 0.019 | 0.029 |
| 127 | N5_27 | WT | T18I | V106D | WT | WT | WT | WT | WT | A446S | 0.005 | 0.013 | 0.015 |
| 128 | N5_28 | AAATCTCGT ACGATAGCAT AAAATCTCGT ACGATAGCAT AAAATCTCGT ACGATGTTAA ATCTCGTACG ATAGCATAAA TCTCGTACGA TAGCACCTGC CCAT | T18I | V106D | WT | WT | H380N | A381G | Y463D | WT | 7.607 | >10.24 | 6.899 |
| 129 | N5_29 | WT | T18I | V106D | WT | WT | WT | WT | WT | A446S | 0.026 | 0.046 | 0.065 |
| 130 | N5_30 | WT | T18I | V106D | WT | WT | WT | WT | WT | A446S | 0.018 | 0.023 | 0.061 |
| 131 | N5_31 | WT | T18I | V106D | WT | WT | WT | WT | WT | A446S | 0.034 | 0.036 | 0.061 |
| 132 | N5_32 | AAATCTCGT ACGATAGCAT AAAATCTCGT ACGATAGCAT AAAATCTCGT ACGATGTTAA ATCTCGTACG ATAGCATAAA TCTCGTACGA TAGCACCTGC CCAT | T18I | V106D | WT | WT | H380N | A381G | Y463D | WT | 5.918 | 8.960 | 6.037 |
| 133 | N5_33 | AAATCTCGT ACGATAGCAT AAAATCTCGT ACGATAGCAT AAAATCTCGT ACGATGTTAA ATCTCGTACG ATAGCATAAA TCTCGTACGA TAGCACCTGC CCAT | T18I | V106D | WT | WT | H380N | A381G | Y463D | WT | 6.851 | 7.410 | 4.996 |
| 134 | N5_34 | WT | T18I | V106D | WT | WT | WT | WT | WT | A446S | 0.018 | 0.022 | 0.056 |
| 135 | N5_35? | ? | ? | ? | ? | ? | ? | ? | ? | ? | 6.799 | >10.24 | 7.111 |
| 136 | N5_36? | ? | ? | ? | ? | ? | ? | ? | ? | ? | 7.664 | >10.24 | 7.233 |
| 137 | N5_37 | WT | T18I | V106D | WT | WT | WT | WT | WT | A446S | 0.034 | 0.029 | 0.072 |
| 138 | N5_38 | AAATCTCGT ACGATAGCAT AAAATCTCGT ACGATAGCAT AAAATCTCGT ACGATGTTAA ATCTCGTACG ATAGCATAAA TCTCGTACGA TAGCACCTGC CCAT | T18I | V106D | WT | WT | H380N | A381G | Y463D | WT | 6.952 | 7.935 | 6.417 |
| 139 | N5_39 | AAATCTCGT ACGATAGCAT AAAATCTCGT ACGATAGCAT AAAATCTCGT ACGATGTTAA ATCTCGTACG ATAGCATAAA TCTCGTACGA TAGCACCTGC CCAT | T18I | V106D | WT | WT | H380N | A381G | Y463D | WT | 6.038 | 6.297 | 5.778 |
| 140 | N5_40 | AAATCTCGT ACGATAGCAT AAAATCTCGT ACGATAGCAT AAAATCTCGT ACGATGTTAA ATCTCGTACG ATAGCATAAA TCTCGTACGA TAGCACCTGC CCAT | T18I | V106D | WT | WT | H380N | A381G | Y463D | WT | 3.378 | 4.699 | 2.404 |
| 141 | N5_41 | AAATCTCGT ACGATAGCAT AAAATCTCGT ACGATAGCAT AAAATCTCGT ACGATGTTAA ATCTCGTACG ATAGCATAAA TCTCGTACGA TAGCACCTGC CCAT | T18I | V106D | WT | WT | H380N | A381G | Y463D | WT | 2.596 | 3.825 | 2.489 |
| 142 | N5_42 | AAATCTCGT ACGATAGCAT AAAATCTCGT ACGATAGCAT AAAATCTCGT ACGATGTTAA ATCTCGTACG ATAGCATAAA TCTCGTACGA TAGCACCTGC CCAT | T18I | V106D | WT | WT | H380N | A381G | Y463D | WT | 2.565 | 4.474 | 2.026 |
| 143 | N5_43 | AAATCTCGT ACGATAGCAT AAAATCTCGT ACGATAGCAT AAAATCTCGT ACGATGTTAA ATCTCGTACG ATAGCATAAA TCTCGTACGA TAGCACCTGC CCAT | T18I | V106D | WT | WT | H380N | A381G | Y463D | WT | 1.810 | 3.084 | 1.288 |
| 144 | N5_44 | AAATCTCGT ACGATAGCAT AAAATCTCGT ACGATAGCAT AAAATCTCGT ACGATGTTAA ATCTCGTACG ATAGCATAAA TCTCGTACGA TAGCACCTGC CCAT | T18I | V106D | WT | WT | H380N | A381G | Y463D | WT | 7.370 | >10.24 | 4.511 |
| 145 | N5_45 | AAATCTCGT ACGATAGCAT AAAATCTCGT ACGATAGCAT AAAATCTCGT ACGATGTTAA ATCTCGTACG ATAGCATAAA TCTCGTACGA TAGCACCTGC CCAT | T18I | V106D | WT | WT | H380N | A381G | Y463D | WT | 6.778 | 8.602 | 3.462 |
| 146 | N5_46 | AAATCTCGT ACGATAGCAT AAAATCTCGT ACGATAGCAT AAAATCTCGT ACGATGTTAA ATCTCGTACG ATAGCATAAA TCTCGTACGA TAGCACCTGC CCAT | T18I | V106D | WT | WT | H380N | A381G | Y463D | WT | 6.768 | 8.069 | 2.540 |
| 147 | N5_47 | AAATCTCGT ACGATAGCAT AAAATCTCGT ACGATAGCAT AAAATCTCGT ACGATGTTAA ATCTCGTACG ATAGCATAAA TCTCGTACGA TAGCACCTGC CCAT | T18I | V106D | WT | WT | H380N | A381G | Y463D | WT | 4.868 | 3.809 | 4.303 |
| 148 | N5_48 | WT | T18I | V106D | WT | WT | WT | WT | WT | A446S | 0.074 | 0.090 | 0.084 |
| 149 | N5_49 | WT | T18I | V106D | WT | WT | WT | WT | WT | A446S | 0.090 | 0.110 | 0.096 |
| 150 | N5_50 | WT | T18I | V106D | WT | WT | WT | WT | WT | A446S | 0.008 | 0.016 | 0.024 |
| 151 | N5_51 | WT | T18I | V106D | WT | WT | WT | WT | WT | A446S | 0.015 | 0.016 | 0.045 |
| 152 | N5_52 | WT | T18I | V106D | WT | WT | WT | WT | WT | A446S | 0.012 | 0.018 | 0.042 |
| 153 | N5_53 | AAATCTCGT ACGATAGCAT AAAATCTCGT ACGATAGCAT AAAATCTCGT ACGATGTTAA ATCTCGTACG ATAGCATAAA TCTCGTACGA TAGCACCTGC CCAT | T18I | V106D | WT | WT | H380N | A381G | Y463D | WT | 2.270 | 5.514 | 1.574 |
| 154 | N5_54 | WT | T18I | V106D | WT | WT | WT | WT | WT | A446S | 0.084 | 0.056 | 0.078 |
| 155 | N5_55? | ? | ? | ? | ? | ? | ? | ? | ? | ? | 1.543 | 5.979 | 1.380 |
| 156 | N5_56 | WT | T18I | V106D | WT | WT | WT | WT | WT | A446S | 0.015 | 0.015 | 0.043 |
| 157 | N5_57 | WT | T18I | V106D | WT | WT | WT | WT | WT | A446S | 0.108 | 0.315 | 0.273 |
| 158 | N5_58 | AAATCTCGT ACGATAGCAT AAAATCTCGT ACGATAGCAT AAAATCTCGT ACGATGTTAA ATCTCGTACG ATAGCATAAA TCTCGTACGA TAGCACCTGC CCAT | T18I | V106D | WT | WT | H380N | A381G | Y463D | WT | 1.250 | 1.673 | 1.672 |
| 159 | N5_59 | WT | T18I | V106D | WT | WT | WT | WT | WT | A446S | 0.039 | 0.070 | 0.064 |
| 160 | N5_60? | ? | ? | ? | ? | ? | ? | ? | ? | ? | 2.497 | 2.415 | 2.751 |
| 161 | N5_61 | AAATCTCGT ACGATAGCAT AAAATCTCGT ACGATAGCAT AAAATCTCGT ACGATGTTAA ATCTCGTACG ATAGCATAAA TCTCGTACGA TAGCACCTGC CCAT | T18I | V106D | WT | WT | H380N | A381G | Y463D | WT | 1.277 | 2.034 | 1.657 |
| 162 | N5_62 | AAATCTCGT ACGATAGCAT AAAATCTCGT ACGATAGCAT AAAATCTCGT ACGATGTTAA ATCTCGTACG ATAGCATAAA TCTCGTACGA TAGCACCTGC CCAT | T18I | V106D | WT | WT | H380N | A381G | Y463D | WT | 0.759 | 0.870 | 1.091 |
| 163 | N5_63 | AAATCTCGT ACGATAGCAT AAAATCTCGT ACGATAGCAT AAAATCTCGT ACGATGTTAA ATCTCGTACG ATAGCATAAA TCTCGTACGA TAGCACCTGC CCAT | T18I | V106D | WT | WT | H380N | A381G | Y463D | WT | 3.711 | 4.724 | 1.836 |
| 164 | N5_64 | WT | T18I | V106D | WT | WT | WT | WT | WT | A446S | 0.094 | 0.087 | 0.080 |
| 165 | N5_65 | WT | T18I | V106D | WT | WT | WT | WT | WT | A446S | 0.074 | 0.069 | 0.094 |
| 166 | N5_66 | WT | T18I | V106D | WT | WT | WT | WT | WT | A446S | 0.087 | 0.070 | 0.076 |
| 167 | N5_67 | WT | T18I | V106D | WT | WT | WT | WT | WT | A446S | 0.013 | 0.015 | 0.034 |
| 168 | N5_68 | AAATCTCGT ACGATAGCAT AAAATCTCGT ACGATAGCAT AAAATCTCGT ACGATGTTAA ATCTCGTACG ATAGCATAAA TCTCGTACGA TAGCACCTGC CCAT | T18I | V106D | WT | WT | H380N | A381G | Y463D | WT | 1.340 | 2.321 | 1.718 |
| 169 | N5_69 | WT | T18I | V106D | WT | WT | WT | WT | WT | A446S | 0.018 | 0.021 | 0.047 |
| 170 | N5_70 | AAATCTCGT ACGATAGCAT AAAATCTCGT ACGATAGCAT AAAATCTCGT ACGATGTTAA ATCTCGTACG ATAGCATAAA TCTCGTACGA TAGCACCTGC CCAT | T18I | V106D | WT | WT | H380N | A381G | Y463D | WT | 6.878 | 8.358 | 4.955 |
| 171 | N5_71 | WT | T18I | V106D | WT | WT | WT | WT | WT | A446S | 0.053 | 0.090 | 0.083 |
| 172 | N5_72 | AAATCTCGT ACGATAGCAT AAAATCTCGT ACGATAGCAT AAAATCTCGT ACGATGTTAA ATCTCGTACG ATAGCATAAA TCTCGTACGA TAGCACCTGC CCAT | T18I | V106D | WT | WT | H380N | A381G | Y463D | WT | 5.592 | 7.162 | 6.173 |
| 173 | N5_73 | AAATCTCGT ACGATAGCAT AAAATCTCGT ACGATAGCAT AAAATCTCGT ACGATGTTAA ATCTCGTACG ATAGCATAAA TCTCGTACGA TAGCACCTGC CCAT | T18I | V106D | WT | WT | H380N | A381G | Y463D | WT | 4.497 | 4.222 | 4.500 |
| 174 | N5_74 | WT | T18I | V106D | WT | WT | WT | WT | WT | A446S | 0.027 | 0.038 | 0.074 |
| 175 | N5_75 | AAATCTCGT ACGATAGCAT AAAATCTCGT ACGATAGCAT AAAATCTCGT ACGATGTTAA ATCTCGTACG ATAGCATAAA TCTCGTACGA TAGCACCTGC CCAT | T18I | V106D | WT | WT | H380N | A381G | Y463D | WT | 5.290 | 4.509 | 5.688 |
| 176 | N5_76 | AAATCTCGT ACGATAGCAT AAAATCTCGT ACGATAGCAT AAAATCTCGT ACGATGTTAA ATCTCGTACG ATAGCATAAA TCTCGTACGA TAGCACCTGC CCAT | T18I | V106D | WT | WT | H380N | A381G | Y463D | WT | 6.004 | 5.679 | 6.047 |
| 177 | N5_77 | AAATCTCGT ACGATAGCAT AAAATCTCGT ACGATAGCAT AAAATCTCGT ACGATGTTAA ATCTCGTACG ATAGCATAAA TCTCGTACGA TAGCACCTGC CCAT | T18I | V106D | WT | WT | H380N | A381G | Y463D | WT | 5.330 | 5.347 | 5.224 |
| 178 | N5_78 | WT | T18I | V106D | WT | WT | WT | WT | WT | A446S | 0.005 | 0.015 | 0.021 |
| 179 | N5_81 | WT | T18I | V106D | WT | WT | WT | WT | WT | A446S | 0.048 | 0.054 | 0.063 |
| 180 | N5_82 | WT | T18I | V106D | WT | WT | WT | WT | WT | A446S | 0.016 | 0.017 | 0.041 |
| 181 | N5_83 | AAATCTCGT ACGATAGCAT AAAATCTCGT ACGATAGCAT AAAATCTCGT ACGATGTTAA ATCTCGTACG ATAGCATAAA TCTCGTACGA TAGCACCTGC CCAT | T18I | V106D | WT | WT | H380N | A381G | Y463D | WT | 2.634 | 6.064 | 3.011 |
| 182 | N5_84 | AAATCTCGT ACGATAGCAT AAAATCTCGT ACGATAGCAT AAAATCTCGT ACGATGTTAA ATCTCGTACG ATAGCATAAA TCTCGTACGA TAGCACCTGC CCAT | T18I | V106D | WT | WT | H380N | A381G | Y463D | WT | 4.754 | 5.864 | 3.780 |
| 183 | N5_85 | WT | T18I | V106D | WT | WT | WT | WT | WT | A446S | 0.080 | 0.094 | 0.088 |
| 184 | N5_86 | AAATCTCGT ACGATAGCAT AAAATCTCGT ACGATAGCAT AAAATCTCGT ACGATGTTAA ATCTCGTACG ATAGCATAAA TCTCGTACGA TAGCACCTGC CCAT | T18I | V106D | WT | WT | H380N | A381G | Y463D | WT | 5.153 | 5.370 | 2.111 |
| 185 | N5_87 | AAATCTCGT ACGATAGCAT AAAATCTCGT ACGATAGCAT AAAATCTCGT ACGATGTTAA ATCTCGTACG ATAGCATAAA TCTCGTACGA TAGCACCTGC CCAT | T18I | V106D | WT | WT | H380N | A381G | Y463D | WT | 6.803 | 8.057 | 6.775 |
| 186 | N5_88 | WT | T18I | V106D | WT | WT | WT | WT | WT | A446S | 0.061 | 0.087 | 0.079 |
| 187 | N5_89 | AAATCTCGT ACGATAGCAT AAAATCTCGT ACGATAGCAT AAAATCTCGT ACGATGTTAA ATCTCGTACG ATAGCATAAA TCTCGTACGA TAGCACCTGC CCAT | T18I | V106D | WT | WT | H380N | A381G | Y463D | WT | 2.449 | 4.478 | 2.317 |
| 188 | N5_90 | WT | T18I | V106D | WT | WT | WT | WT | WT | A446S | 0.018 | 0.020 | 0.055 |
| 189 | N5_91 | AAATCTCGT ACGATAGCAT AAAATCTCGT ACGATAGCAT AAAATCTCGT ACGATGTTAA ATCTCGTACG ATAGCATAAA TCTCGTACGA TAGCACCTGC CCAT | T18I | V106D | WT | WT | H380N | A381G | Y463D | WT | 3.130 | 4.263 | 3.355 |
| 190 | N5_92 | WT | T18I | V106D | WT | WT | WT | WT | WT | A446S | 0.029 | 0.036 | 0.074 |
| 191 | N5_93 | AAATCTCGT ACGATAGCAT AAAATCTCGT ACGATAGCAT AAAATCTCGT ACGATGTTAA ATCTCGTACG ATAGCATAAA TCTCGTACGA TAGCACCTGC CCAT | T18I | V106D | WT | WT | H380N | A381G | Y463D | WT | 1.491 | 4.167 | 3.211 |
| 192 | N5_94 | WT | T18I | V106D | WT | WT | WT | WT | WT | A446S | 0.018 | 0.024 | 0.062 |
| 193 | N5_95 | AAATCTCGT ACGATAGCAT AAAATCTCGT ACGATAGCAT AAAATCTCGT ACGATGTTAA ATCTCGTACG ATAGCATAAA TCTCGTACGA TAGCACCTGC CCAT | T18I | V106D | WT | WT | H380N | A381G | Y463D | WT | 2.557 | 3.482 | 2.635 |
| 194 | N5_96 | AAATCTCGT ACGATAGCAT AAAATCTCGT ACGATAGCAT AAAATCTCGT ACGATGTTAA ATCTCGTACG ATAGCATAAA TCTCGTACGA TAGCACCTGC CCAT | T18I | V106D | WT | WT | H380N | A381G | Y463D | WT | 6.681 | 7.430 | 5.895 |
| 195 | N5_97 | WT | T18I | V106D | WT | WT | WT | WT | WT | A446S | 0.065 | 0.077 | 0.107 |
| 196 | N5_98 | AAATCTCGT ACGATAGCAT AAAATCTCGT ACGATAGCAT AAAATCTCGT ACGATGTTAA ATCTCGTACG ATAGCATAAA TCTCGTACGA TAGCACCTGC CCAT | T18I | V106D | WT | WT | H380N | A381G | Y463D | WT | 7.336 | 9.459 | 7.229 |
| 197 | N5_99? | ? | ? | ? | ? | ? | ? | ? | ? | ? | 5.599 | 6.688 | 2.938 |
| 198 | N5_100 | AAATCTCGT ACGATAGCAT AAAATCTCGT ACGATAGCAT AAAATCTCGT ACGATGTTAA ATCTCGTACG ATAGCATAAA TCTCGTACGA TAGCACCTGC CCAT | T18I | V106D | WT | WT | H380N | A381G | Y463D | WT | 5.702 | 4.959 | 5.381 |
| 199 | N5_101 | AAATCTCGT ACGATAGCAT AAAATCTCGT ACGATAGCAT AAAATCTCGT ACGATGTTAA ATCTCGTACG ATAGCATAAA TCTCGTACGA TAGCACCTGC CCAT | T18I | V106D | WT | WT | H380N | A381G | Y463D | WT | 2.803 | 3.863 | 3.281 |
| 200 | N5_102 | WT | T18I | V106D | WT | WT | WT | WT | WT | A446S | 0.030 | 0.028 | 0.069 |
