# Supplementary material for: Pfcyp51 exclusively determines reduced sensitivity to 14α-demethylase inhibitor fungicides in the banana black Sigatoka pathogen Pseudocercospora fijiensis
Source: PLoS One. 2019 Oct 17;14(10):e0223858. doi: 10.1371/journal.pone.0223858 (PMC6797121; doi:10.1371/journal.pone.0223858)
Supplement: S3 Table — 1The information of the genes was collected from the Joint Genome Institute, Mycosphaerella fijiensis (Pseudocercospora fijiensis) v2.0 portal. https://mycocosm.jgi.doe.gov/pages/search-for-genes.jsf?organism=Mycfi2. (DOCX) [file pone.0223858.s008.docx]

| **Transcript ID:** | **Location:** | **Gene ontology** | **Definition** |
| --- | --- | --- | --- |
| 177154 | scaffold_7:1909687-1917514 (+) | Putative protein | No annotation yet |
| 177158 | scaffold_7:1938540-1939146 (-) | Putative protein | No annotation yet |
| 31195 | scaffold_7:1967582-1967875 (+) | Putative protein | No annotation yet |
| 31837 | scaffold_7:1920971-1921141(+) | Putative protein | No annotation yet |
| 22691 | scaffold_7:1949446-1950451 (-) | GO:0004553 hydrolase activity, hydrolyzing O-glycosyl compounds. Catalysis of the hydrolysis of any O-glycosyl bond. | No annotation yet |
| 189819 | scaffold_7:1929097-1930398 (-) | GO:0003824 catalytic activities. Catalysis of a biochemical reaction at physiological temperatures. | No annotation yet |
| 30851 | scaffold_7:1959643-1961193 (-) | Putative protein | No annotation yet |
| 101452 | scaffold_7:1931010-1931678 (-) | Putative protein | No annotation yet |
| 101452 | scaffold_7:1931010-1931678 (-) | Putative protein | No annotation yet |
| 211990 | scaffold_7:1943313-1944619 (-) | GO:0000172 ribonuclease MRP complex. A ribonucleoprotein complex that contains an RNA molecule of the snoRNA family, and cleaves the rRNA precursor as part of rRNA transcript processing. It also has other roles: In S. cerevisiae it is involved in cell cycle-regulated degradation of daughter cell-specific mRNAs, while in mammalian cells it also enters the mitochondria and processes RNAs to create RNA primers for DNA replication. | No annotation yet |
| 7504 | scaffold_7:1939638-1940268 (+) | Putative protein | No annotation yet |
| 77954 | scaffold_7:1945205-1946623 (-) | GO:0004553 hydrolase activity, hydrolyzing O-glycosyl compounds. Catalysis of the hydrolysis of any O-glycosyl bond. | No annotation yet |
| 31273 | scaffold_7:1947348-1948031 (-) | GO:0004733 pyridoxamine-phosphate oxidase activity. Catalysis of the reaction: pyridoxamine 5'-phosphate + H2O + O2 = pyridoxal 5'-phosphate + NH3 + hydrogen peroxide. | No annotation yet |
| 198484 | scaffold_7:1951727-1952825 (-) | Putative protein | No annotation yet |
| 77958 | scaffold_7:1953723-1954161 (-) | Putative protein | No annotation yet |
| 77959 | scaffold_7:1954646-1955608 (+) | Putative protein | No annotation yet |
| 198487 | scaffold_7:1955999-1956492 (+) | Putative protein | No annotation yet |
| 204493 | scaffold_7:1957874-1958706 (+) | Putative protein | No annotation yet |
| 77962 | scaffold_7:2016030-2016929 (-) | Putative protein | No annotation yet |
| 177166 | scaffold_7:2021508-2023032 (+) | Putative protein | No annotation yet |
| 177167 | scaffold_7:2026479-2029163 (+) | Putative protein | No annotation yet |
| 177169 | scaffold_7:2032413-2034139 (+) | Putative protein | No annotation yet |
| 77963 | scaffold_7:2035236-2035724 (+) | Putative protein | No annotation yet |
| 77964 | scaffold_7:2036475-2038341 (-) | Putative protein | No annotation yet |
| 208509 | scaffold_7:2038381-2039739 (+) | Putative protein | No annotation yet |
| 155993 | scaffold_7:2039032-2039971 (-) | GO:0003723 RNA binding. Interacting selectively and non-covalently with an RNA molecule or a portion thereof. | No annotation yet |
| 49844 | scaffold_7:2040187-2041265 (+) | GO:0004298 threonine-type endopeptidase activity. Catalysis of the hydrolysis of internal peptide bonds in a polypeptide chain by a mechanism in which the hydroxyl group of a threonine residue at the active center acts as a nucleophile. | No annotation yet |
| 96804 | scaffold_6:4257330-4259091 (-) | GO:0003677 DNA binding. Any molecular function by which a gene product interacts selectively with DNA (deoxyribonucleic acid). GO:0003700 sequence-specific DNA binding transcription factor activity. Interacting selectively and non-covalently with a specific DNA sequence in order to modulate transcription. The transcription factor may or may not also interact selectively with a protein or macromolecular complex. GO:0006355 regulation of transcription, DNA-dependent. Any process that modulates the frequency, rate or extent of cellular DNA-dependent transcription. | No annotation yet |
| 166575 | scaffold_7:2042679-2044011 (+) | GO:0005515 protein binding. Interacting selectively and non-covalently with any protein or protein complex (a complex of two or more proteins that may include other non-protein molecules). | No annotation yet |
| 77971 | scaffold_7:2049509-2051776 (-) | Putative protein | No annotation yet |
| 31107 | scaffold_7:2054261-2055433 (+) | GO:0003824 catalytic activity. Catalysis of a biochemical reaction at physiological temperatures. In biologically catalyzed reactions, the reactants are known as substrates, and the catalysts are naturally occurring macromolecular substances known as enzymes. Enzymes possess specific binding sites for substrates, and are usually composed wholly or largely of protein, but RNA that has catalytic activity (ribozyme) is often also regarded as enzymatic. | No annotation yet |
| 204500 | scaffold_7:2056817-2057682 (+) | Putative protein | No annotation yet |
| 156006 | scaffold_7:2058401-2059893 (-) | GO:0000166 nucleotide binding. Interacting selectively and non-covalently with a nucleotide, any compound consisting of a nucleoside that is esterified with (ortho)phosphate or an oligophosphate at any hydroxyl group on the ribose or deoxyribose moiety. | No annotation yet |
| 49849 | scaffold_7:2060248-2062273 (+) | GO:0000015 phosphopyruvate hydratase complex. A multimeric enzyme complex, usually a dimer or an octamer, that catalyzes the conversion of 2-phospho-D-glycerate to phosphoenolpyruvate and water. | No annotation yet |
| 208514 | scaffold_7:2044371-2048954 (-) | GO:0008060 ARF GTPase activator activity. Increases the rate of GTP hydrolysis by the GTPase ARF. | No annotation yet |
| 77972 | scaffold_7:2052410-2053032 (-) | Putative protein | No annotation yet |
| 31190 | scaffold_7:2055461-2056561 (-) | GO:0003824 catalytic activity. Catalysis of a biochemical reaction at physiological temperatures. In biologically catalysed reactions, the reactants are known as substrates, and the catalysts are naturally occurring macromolecular substances known as enzymes. Enzymes possess specific binding sites for substrates, and are usually composed wholly or largely of protein, but RNA that has catalytic activity (ribozyme) is often also regarded as enzymatic. | No annotation yet |
| 211997 | scaffold_7:2062352-2063340 (-) | Putative protein | No annotation yet |
| 198503 | scaffold_7:2063728-2064756 (+) | Putative protein | No annotation yet |
| 31199 | scaffold_7:2082954-2084195 (-) | GO:0006508 proteolysis. The chemical reactions and pathways resulting in the breakdown of a protein by the destruction of the native, active configuration, with the hydrolysis of peptide bonds. | No annotation yet |
| 156013 | scaffold_7:2084798-2087077 (+) | GO:0004672 protein kinase activity. Catalysis of the phosphorylation of an amino acid residue in a protein, usually according to the reaction: a protein + ATP = a phosphoprotein + ADP. | No annotation yet |
| 77984 | scaffold_7:2088199-2089353 (-) | Putative protein | No annotation yet |
| 211999 | scaffold_7:2096114-2096935 (-) | Putative protein | No annotation yet |
| 63438 | scaffold_7:2122388-2122960 (+) | Putative protein | No annotation yet |
| 104048 | scaffold_7:2090006-2095772 (-) | GO:0005488 binding. The selective, non-covalent, often stoichiometric, interaction of a molecule with one or more specific sites on another molecule. | No annotation yet |
| 156019 | scaffold_7:2115877-2116567 (+) | Putative protein | No annotation yet |
| 212000 | scaffold_7:2117112-2119076 (+) | Putative protein | No annotation yet |
| 204508 | scaffold_7:2117112-2119587 (-) | Putative protein | No annotation yet |
| 30715 | scaffold_7:2119919-2121685 (-) | GO:0004497 monooxygenase activity. Catalysis of the incorporation of one atom from molecular oxygen into a compound and the reduction of the other atom of oxygen to water. GO:0005506 iron ion binding. Interacting selectively and non-covalently with iron (Fe) ions. GO:0006118 electron transport. OBSOLETE. The transport of electrons from an electron donor to an electron acceptor. GO:0020037 heme binding. Interacting selectively and non-covalently with heme, any compound of iron complexed in a porphyrin (tetrapyrrole) ring. | Lanosterol 14α-demethylase, catalyses the C-14 demethylation of lanosterol in the ergosterol biosynthesis pathway. Target for azole fungicides (Rafael Arango, 2008-11-10). Name: CYP51 (Rafael Arango, 2008-11-10) |
| 63438 | scaffold_7:2122388-2122960 (+) | Putative protein | No annotation yet |
| 212002 | scaffold_7:2124095-2125034 (+) | Putative protein | No annotation yet |
| 86816 | scaffold_7:2125667-2128069 (+) | GO:0005215 transporter activity. Enables the directed movement of substances (such as macromolecules, small molecules, ions) into, out of or within a cell, or between cells. GO:0006810 transport. The directed movement of substances (such as macromolecules, small molecules, ions) into, out of or within a cell, or between cells, or within a multicellular organism by means of some agent such as a transporter or pore. GO:0016020 membrane. Double layer of lipid molecules that encloses all cells, and, in eukaryotes, many organelles; may be a single or double lipid bilayer; also includes associated proteins. | No annotation yet |
| 77993 | scaffold_7:2128129-2130717 (-) | Putative protein | No annotation yet |
